# Supplementary material for: Single-molecule FRET probes allosteric effects on protein-translocating pore loops of a AAA+ machine
Source: Biophys J. 2024 Jan 9;123(3):374–88. doi: 10.1016/j.bpj.2024.01.002 (PMC10870172; doi:10.1016/j.bpj.2024.01.002)
Supplement: Document S2. Article plus supporting material [file mmc5.pdf]

Biophysical Journal
Article

Single-molecule FRET probes allosteric effects on protein-translocating pore loops of a AAA+ machine

Marija Iljina,[1] Hisham Mazal,[1] Ashan Dayananda,[2] Zhaocheng Zhang,[2] George Stan,[2,*] Inbal Riven,[1] and Gilad Haran[1,*]
[1]Department of Chemical and Biological Physics, Weizmann Institute of Science, Rehovot, Israel and [2]Department of Chemistry, University of Cincinnati, Cincinnati, Ohio

ABSTRACT AAA+ proteins (ATPases associated with various cellular activities) comprise a family of powerful ring-shaped ATP-dependent translocases that carry out numerous vital substrate-remodeling functions. ClpB is a AAA+ protein disaggregation machine that forms a two-tiered hexameric ring, with flexible pore loops protruding into its center and binding to substrate proteins. It remains unknown whether these pore loops contribute only passively to substrate-protein threading or have a more active role. Recently, we have applied single-molecule FRET spectroscopy to directly measure the dynamics of substrate-binding pore loops in ClpB. We have reported that the three pore loops of ClpB (PL1-3) undergo large-scale fluctuations on the microsecond timescale that are likely to be mechanistically important for disaggregation. Here, using single-molecule FRET, we study the allosteric coupling between the pore loops and the two nucleotide-binding domains of ClpB (NBD1–2). By mutating the conserved Walker B motifs within the NBDs to abolish ATP hydrolysis, we demonstrate how the nucleotide state of each NBD tunes pore-loop dynamics. This effect is surprisingly long-ranged; in particular, PL2 and PL3 respond differentially to a Walker B mutation in either NBD1 or NBD2, as well as to mutations in both. We characterize the conformational dynamics of pore loops and the allosteric paths connecting NBDs to pore loops by molecular dynamics simulations and find that both principal motions and allosteric paths can be altered by changing the ATPase state of ClpB. Remarkably, PL3, which is highly conserved in AAA+ machines, is found to favor an upward conformation when only NBD1 undergoes ATP hydrolysis but a downward conformation when NBD2 is active. These results explicitly demonstrate a significant long-range allosteric effect of ATP hydrolysis sites on pore-loop dynamics. Pore loops are therefore established as active participants that undergo ATP-dependent conformational changes to translocate substrate proteins through the central pores of AAA+ machines.

SIGNIFICANCE Molecular machines function by coupling ATP hydrolysis to mechanical motion. How this coupling occurs and what timescales are involved remains an open question. Here, we use single-molecule FRET spectroscopy to measure the real-time dynamics of pore loops, which are essential protein-translocating elements of the ATP-dependent disaggregation machine ClpB. Using a series of mutations of the ATP-hydrolysis motifs of ClpB, we find that the motions of these pore loops take place on the microsecond timescale, yet they are markedly affected by the much slower changes in the nucleotide state of the machine. Generally, this study shows that protein machines, such as ClpB, are wired to harness ATP binding and hydrolysis to allosterically affect distal events, such as function-related pore-loop mechanics.

INTRODUCTION

Members of the AAA+ (ATPases associated with various cellular activities) protein family are diverse molecular machines that perform multiple ATP-dependent biological functions in cells (1,2). In their functional forms, these proteins typically assemble into asymmetric hexameric rings that can translocate client substrate DNA (3,4) or substrate proteins through their central pore (5,6). The residues that

Submitted May 2, 2023, and accepted for publication January 2, 2024.

*Correspondence: stangg@ucmail.uc.edu or gilad.haran@weizmann.ac.il

Hisham Mazal's present Address is Max Planck Institute for Science of Light, Staudtstrasse 2, 90158 Erlangen, Germany.

Editor: Judy Kim.

https://doi.org/10.1016/j.bpj.2024.01.002

© 2024 Biophysical Society.

This is an open access article under the CC BY license (http://creativecommons.org/licenses/by/4.0/).

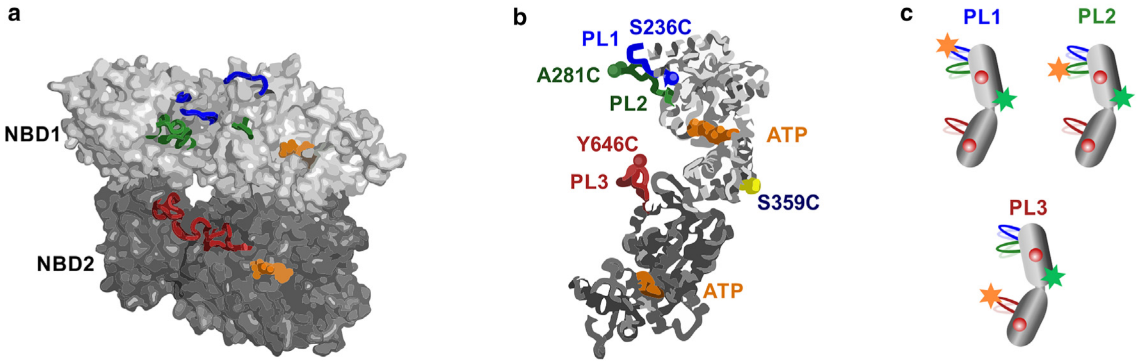

FIGURE 1 Location of pore loops in ClpB hexamer and fluorescence labeling scheme for single-molecule experiments. (*a*) Side view of ClpB hexamer from *E. coli* (PDB: 6OAX) (9), with three protomers removed to reveal the central pore. The topmost N-terminal domain is absent in this structure. Nucleotides at NBD1 and NBD2, bound at the subunit interfaces, are shown in orange. PL1, PL2, and PL3 of the three shown protomers (A, B, and F) are colored in blue, green and red, respectively. (*b*) Monomer structure of ClpB (PDB: 6OAX, protomer A (9)). Pore loop 1 (PL1) residues 235–245, corresponding to the sequence GSLLAGA**KYRG**, are shown in blue. Pore loop 2 (PL2) residues 272–290 (LHTVV**GAG**KAEGAVDAGNM) are in green. Pore loop 3 (PL3) residues 637–650 (IGAPP**GYVG**YEEGG) are in red. Numbering and the primary sequence are as in full-length *T. thermophilus* (TT) ClpB, used in all experiments in this work. Conserved residues of the primary sequence are in bold. (A short additional pore loop adjacent to PL3, identified in the PDB 6OAX (9) structure, was found to be too close to PL3 and was not studied here.) Residues S236C, A281C, and Y646C, used for fluorescence labeling of the pore loops, are shown as spheres on PL1, PL2, and PL3, whereas residue S359C, used as a reference position in FRET assays, is shown in yellow. Residue numbers are from TT ClpB. (*c*) Cartoon representation of the fluorescence double-labeling scheme to study PLs showing the positions of two Alexa Fluor dyes (as stars) within ClpB monomer. Red spheres represent ATP bound to the two NBDs of ClpB. PL1 construct S236C-S359C, PL2 construct A281C-S359C, and PL3 construct S359C-Y646C. In all experiments, we use N-terminally truncated TT ClpB. This and all the subsequent figures are available in color online. To see this figure in color, go online.

constitute ATPase pockets, located at the protomer interfaces (Fig. 1 *a*), are the highly conserved Walker A motif, GXXXXGK[T/S] (where X is any amino acid), involved in ATP binding, and the Walker B motif, hhhD[D/E] (where h is a hydrophobic residue), essential for ATP hydrolysis (7). ClpB is a bacterial AAA+ disaggregase that has two nucleotide-binding domains, NBD1 and NBD2, within each protomer (8) and forms a two-tiered hexameric ring (9). Like other members of the AAA+ family, it binds its substrate proteins by a set of pore loops lining the central pore, including pore loop 1 and pore loop 2 (PL1 and PL2) located in NBD1 and pore loop 3 (PL3), located in NBD2 (9,10) (Fig. 1 *a* and *b*).

ATP-dependent substrate-protein translocation across the central pore of AAA+ machines is thought to be driven by large-scale rearrangements of individual protomers within the hexameric rings (1,2), although the precise mechanism still remains debatable. Multiple recent cryoelectron microscopy (cryo-EM) reconstructions of substrate-bound AAA+ hexamers suggested a structure in which the protomers within the rings are mobile (2) and generally show a spiral arrangement. Consistent with these structures, substrate-protein translocation is proposed to occur after sequential ATP hydrolysis in a "hand-over-hand" mechanism. In this mechanism, upon ATP hydrolysis and re-binding, subunits within a hexamer move one by one, resulting in a unidirectional translocation of the substrate protein across the central pore with a uniform step size of two residues (3,4). This mechanism is proposed to describe the operation of increasingly more complex AAA+ systems (11). However, the sequential ATP hydrolysis and its associated hand-over-hand translocation mechanism might not be applicable to all AAA+ machines, and other models, concerted and probabilistic ATP hydrolysis, were also put forward for several systems. For example, structural analysis of the hexameric helicase LTag suggested a concerted nucleotide binding and exchange mechanism (12). Furthermore, two relatively recent cryo-EM studies of substrate-bound ClpXP yielded highly similar hexameric staircase structures but two different interpretations were offered, and either a sequential (13) or a probabilistic mechanism was proposed (14,15). Biochemical studies on covalently tethered ClpX (16) and HslU (17) found that these AAA+ hexamers remained functional even with multiple inactive subunits, supporting a probabilistic mechanism. In addition, single-molecule optical tweezers assays with ClpXP (18) and ClpB (19) and high-speed atomic force microscopy experiments with Abo1 (20) yielded results that were more consistent with probabilistic mechanisms.

Pore loops lining the central channel of AAA+ proteins are essential substrate-binding elements, and their mutations severely impair translocation activities of these machines (2). PL1 and PL3 in ClpB contain conserved residues KYRG and GYVG, respectively (8) (Fig. 1 *b*). The highly conserved functional tyrosines are well known to bind to client proteins in ClpB (6,21) and in other AAA+ machines (22,23). Furthermore, structural analyses revealed that, in these pore loops, flanking hydrophobic amino acids are also in contact with substrate proteins (10,14). Not only the primary sequence but also the spatial arrangement of the substrate-binding pore loops within AAA+ hexamers is remarkably well conserved. They form an almost

identical spiral-staircase pattern around the bound substrate in a vast number of cryo-EM structures of AAA+ hexamers, including Yme1, spastin, proteasome, Cdc48, and NSF (2). The primary sequence of PL2 is not well conserved, although its residues were also found to be in contact with protein substrates in ClpB (9,24) and in its yeast analogue Hsp104 (10). Furthermore, its functional significance for disaggregation was verified by mutational analysis (10).

Interestingly, increasing amounts of experimental evidence indicate that conformational changes of the substrate-binding pore loops contribute to the ATP-dependent translocation by AAA+ machines. Indeed, based on monitoring fluorescence from a tryptophan mutant of pore loop 1 in ClpB, it was suggested that this pore loop undergoes conformational changes that depend on the type of bound nucleotide (21). Furthermore, based on disulfide crosslinking of substrate peptides to pore loops in ClpX (25) and on mutational and functional analyses (26), it was proposed that the pore loops undergo ATP-dependent structural changes between "up" and "down" conformations along the axial channel to propel the bound substrate-protein through the hexameric ring. These bulk biochemical studies could not yield a structural model to describe the detected conformational changes. More recently, however, several structural studies explicitly visualized distinct conformational states of the pore loops. From analysis of the structures, it could be inferred that the GYVG pore loops in ClpB and ClpXP respond to substrate-protein binding (13,24). Moreover, PL2 was captured in distinct up and down conformations in the crystal structure of ClpB's yeast analogue Hsp104 (10).

Although recent advances in structural methods have provided unprecedented details on substrate-protein binding by AAA+ proteins and even on the conformational changes within their pore loops, the extent and timescales of these motions and their potential coupling to the ATPase activity of these proteins remain to be characterized. In particular, the relationship between the nucleotide state of individual protomers and the conformations of their pore loops are challenging to study due to averaging of the nucleotide-binding sites in cryo-EM (2). Moreover, detecting the real-time dynamics of distinct pore-loop types during the ATP-dependent activity of AAA+ proteins under native conditions in aqueous solution requires suitable experimental techniques. Recently, we employed a powerful combination of single-molecule FRET (smFRET) spectroscopy and photon-by-photon hidden Markov modeling (27) to study the dynamics of pore loops in ClpB under conditions of active ATP hydrolysis (28). We found that these pore loops fluctuate on the microsecond timescale between two major conformations, up and down, along the central pore and that the populations of these two conformations change upon binding to substrate-protein. Furthermore, we found that the dynamics of PL2 and PL3 respond to ATP hydrolysis, and the dynamics of PL1 and PL3 are correlated to the bulk disaggregation activity of ClpB. These differential responses led us to propose that the pore loops act as Brownian ratchets to translocate substrate proteins through the central channel. In particular, we suggested that the ATP-dependent PL2 and PL3 both serve as pawls that rectify substrate-protein translocation through ClpB's central channel. Here, we set for the first time to investigate the relationship between the nucleotide state of each of the two NBDs of a ClpB hexamer and pore-loop dynamics. To this end, we introduce mutations into the conserved Walker A and Walker B motifs in either NBD1 or NBD2 or both to restrict ClpB to pre-defined ATP-activity states, and we characterize these constructs by smFRET spectroscopy. Furthermore, we study the potential coupling between the NBDs and the pore loops by molecular dynamics (MD) simulations to gain residue-level insights. We demonstrate that the nucleotide states of NBD1 and NBD2 separately can alter the dynamics of all three sets of pore loops, and PL2 and PL3 experience the most prominent modulations. Thus, our results reveal unexpected cooperative allosteric interactions between both ATP-binding sites and the pore loops of ClpB, and they shed light on how ATP binding and hydrolysis drive substrate-protein translocation by the machine.

MATERIALS AND METHODS

Protein expression and purification

All mutant proteins were generated through standard site-directed mutagenesis. *Thermus thermophilus* ClpB variant with truncated N-terminal domain, starting at residue 141 (Val), (referred to as ClpB throughout), and its mutants, cloned into a pET28b vector, were expressed in *Escherichia coli* and purified as described previously (28,29). The truncated ClpB was selected to avoid any hindrance to the fluorescent dye on PL1 by the N domain and was previously verified to be fully assembled and functional (28).

Protein labeling and protomer mixing

Double-cysteine mutants of ClpB were labeled with Alexa Fluor 488 C5 maleimide (AF488) and Alexa Fluor 594 C5 maleimide (AF594) dyes (Thermo Fisher) following a protocol described in our previous work (28). Any unreacted dye molecules were removed on a desalting column (Sephadex G25, GE Healthcare). For the preparation of ClpB hexamers with only one labeled subunit, suitable for smFRET measurements, AF488-AF594-labeled double-cysteine mutants were combined with 100-fold molar excess of unlabeled cysteine-less ClpB. With this ratio, the probability for the incorporation of one labeled protomer in a hexamer was 5.7%, whereas the probability to find two labeled protomers in the same hexamer was as low as 0.15%. To achieve full refolding and homogeneous reassembly, the protein solutions were initially dialyzed in the presence of 6 M GdmCl. This was followed by dialysis steps in the presence of 4, 2, 1, and 0 M GdmCl. The final steps involved extensive dialysis into low-salt buffer (25 mM HEPES, 25 mM KCl, 10 mM $MgCl_2$, 2 mM ATP, pH 8) and filtration through 0.1-μm filters (Whatman Anotop-10). The assembled ClpB was aliquoted, flash-frozen, and stored at -80°C until further use. For the preparation of hexamers containing a functional mutation (any of the Walker mutants described in this study), fluorescently double-labeled ClpB bearing the mutation was mixed with the 100-fold excess of unlabeled

cysteine-less ClpB containing the same functional mutation and reassembled following the above procedure.

ATPase activity measurements

ATP activity of ClpB mutants was measured using a coupled colorimetic assay (30). ClpB or its mutants (1 μM total monomer concentration) were incubated in the presence of 2 mM ATP in 50 mM HEPES (pH 8), 50 mM KCl, and 0.01% Tween 20, with an ATP regeneration system (2.5 mM phosphoenol pyruvate, 10 units/mL pyruvate kinase, 15 units/mL lactate dehydrogenase, 2 mM 1,4 dithioerythritol, 2 mM EDTA, and 0.25 mM NADH). To assess the effect of the model substrate κ-casein (Sigma Aldrich), it was added to a final concentration of 25 μM. ATP hydrolysis was initiated by adding $MgCl_2$ (10 mM) and measured by monitoring the decrease in NADH absorption over time at 340 nm using a microplate reader (Synergy HTX, BioTek) equilibrated at 25°C. The rate of ATP hydrolysis was determined from the initial linear slope of the measured data. ATP hydrolysis rate per ClpB monomer per minute is reported.

smFRET measurements

Custom-made glass flow chambers for smFRET measurements were prepared as previously reported (31). The chambers were coated with a supported lipid bilayer composed of egg phosphatidylcholine (Avanti Polar Lipids) to prevent protein absorption to the glass surface. The reassembled hexamers of ClpB were diluted to ~50 pM labeled ClpB, which corresponds to ~5 nM total ClpB; added into buffer (25 mM HEPES, 25 mM KCl, 10 mM $MgCl_2$, 2 mM ATP, 0.01% Tween 20, pH 8); loaded into the chambers; and sealed with silicon grease. Experiments in the absence of ATP and Mg^{2+} (Fig. S3) were performed by simply omitting these chemicals from the solution; it is possible that residual ATP/Mg^{2+} might have still remained in these samples from the protein purification procedures, but their quantities were too small to affect the proteins. Measurements on freely diffusing molecules were conducted as described before (28), using a MicroTime 200 confocal fluorescence microscope (PicoQuant). The samples were excited using a pulsed interleaved excitation scheme with 485- and 594-nm diode lasers pulsed at a 3:1 ratio, with the repetition rate of 40 MHz, operating at 50 and 8 μW, respectively. The emitted photons were split into two channels by a dichroic mirror (FF580-FDi01, Semrock), and passed through band-pass filters (520/35 nm, BrightLine, Semrock, for the AF488 emission and ET-645/75m, Chroma, for the AF594 emission). Photon arrival times were detected by two single-photon avalanche photo-diodes (Excelitas SPCM-AQR-14-TR) coupled to a standalone time-correlated single-photon-counting module (HydraHarp 400, PicoQuant). Data were acquired for around 5 h per sample at a fixed ambient temperature (22°C), and no evidence of any temperature fluctuations or heating effects was observed. At least two samples were analyzed per condition.

smFRET data analysis and H^{2}MM analysis

Fluorescence bursts, corresponding to the single molecules of ClpB, were selected using data analysis workflows developed in the lab (27–29,31). A cutoff of 5 μs was used to effectively separate fluorescence bursts from the background. Raw FRET efficiency and raw stoichiometry values were calculated as described (32), and a two-dimensional histogram of raw stoichiometry against raw FRET efficiency was constructed and used to calculate correction factors, the leak (~0.05), and the direct excitation (~0.02). After data correction using these factors, FRET efficiency for each burst was calculated (as photons arriving from the acceptor channel divided by the total number of photons). To obtain the final corrected FRET histogram without the donor-only and acceptor-only populations, we selected only photon bursts with a stoichiometry corresponding to molecules bearing both active donor and acceptor dyes and containing at least 30 photons. The same parameters for the selection of double-labeled molecules were used throughout. These stringent selection criteria eliminated any potential photobleaching/blinking events. Once the double-labeled molecules were selected, normalized FRET efficiency distribution functions were displayed as histograms with 35 bins. Note that FRET efficiency distributions were used only for qualitative comparison.

For the quantitative kinetic analysis, we used the same selected data for a maximum likelihood hidden Markov modeling analysis, H^{2}MM, introduced and described in detail previously (27). In this analysis, the arrival time and the type (donor or acceptor) of the selected photons represent the observation sequence (O). The model (λ) comprises 1) the probability matrix (Π), 2) the transition matrix (A), and 3) the observation matrix (B). The algorithm performs the following steps: 1) initialization, when initial model parameters Π, A, and B are guessed (using 50 initial guesses). 2) Expectation, when the model parameters Π, A, and B are learned given the observation sequence O. This is an iterative step and proceeds using the forward-backward Baum-Welch algorithm (a special case of the Expectation-Maximization algorithm). 3) Maximization, where Π, A, and B are re-calculated based on estimators derived from the previous step. 4) Determination of the model's likelihood, P(O|λ), defined as the conditional probability of observing the sequence O given the model λ. 5) Viterbi algorithm: using the best model ($\lambda = (\Pi, A, B)$, which is characterized with the highest likelihood across all 50 guesses, the algorithm calculates the most probable sequence of states. For the H^{2}MM analysis, we chose ~7000 photon trajectories per sample and analyzed them with a two-state model, where the number and FRET efficiency of the states are fixed (detailed in Supporting material, section "supplemental data analysis details" and Table S1), and other parameters are freely and independently optimized. The choice of the fixed two-state model was based on our preceding finding that the free-energy profiles derived from pore-loop data displayed two minima (28). The approach was validated by recoloring and segmentation analyses as well as dwell-time calculations (detailed in Supporting material, section "supplemental data analysis details"; Fig. S2; Table S6). Effective equilibrium coefficient, defined as $K_i = \frac{k^i_{21}}{k^i_{12}} = \frac{P^i_1}{P^i_2}$ (where $k^i{}_{lm}$ is the transition rate from state l to state m and $P^i{}_l$ is the population of state l for pore loop i) was derived from the H^{2}MM analysis of each pore-loop type (Table S6).

MD simulations

We performed MD simulations of wild-type and double-Walker B (BB) mutant of the *E. coli* ClpB using the Gromacs 2022 package (33) and the GROMOS96 54A7 force field. ClpB structures, truncated at N-terminal domains (amino acids 1–160) and middle domains (amino acids 409–524), were modeled using the cryo-EM structure corresponding to the pre-hydrolysis configuration (PDB: 6OAX (9)). We used the Modeller software version 9.23 (34) to build missing loop residues of the pre-hydrolysis conformer, namely amino acid residues 525–528 in protomer 5 and 284–293 in protomer 6 (using protomer numbering in accord with the cryo-EM studies (9)). To ensure chain connectivity in the absence of middle domains, in each chain, residues 408 and 525 were connected through a linker comprising five Gly residues. In the pre-hydrolysis state, protomer 1 is bound to ATPγS in NBD1 and to ADP in NBD2, protomers 2 through 5 are bound to ATPγS in both NBD1 and NBD2, and protomer 6 is bound to ADP in both NBD1 and NBD2. In our simulations, ATPγS and ADP molecules were modeled by using the Automated Topology Builder server, which generates force-field parameters compatible with the GROMOS96 54A7 force field (35). Wild-type ClpB simulations used the same setup and extended the timescales probed in our previous studies (36). ClpB mutants were modeled by using PyMOL (35) to implement Walker B mutations in the cryo-EM structure, E279A in NBD1, and E678A in NBD2. These MD simulations, principal component analyses (PCAs), and analyses of optimal and suboptimal paths are described in detail in the Supporting material (section "supplemental computational methods").

Double-mutant cycles for Walker B mutants

Double-mutant cycles (DMCs) were constructed as previously described (37). More specifically, the free energy, ΔG_i in Jmol^{-1}, for each PL mutant was calculated from equilibrium coefficients according to:

$\Delta G_i = -RTln(K_i)$ where R = 8.314 $\mathrm{Jmol}^{-1}\mathrm{K}^{-1}$ and T = 295.15 K. Subsequently, the free-energy change associated with a mutation (from wild type to B1 or from B2 to BB) was calculated as follows:

$$\Delta\Delta G_{iWT\rightarrow B1} = \Delta G_{iWT} - \Delta G_{iB1}$$

$$\Delta\Delta G_{iB2\rightarrow BB} = \Delta G_{iB2} - \Delta G_{iBB}$$

and plotted along the edges of the cycles (Figs. 6 *d–f* and S15).

In the DMCs analysis, if the $\Delta\Delta G_i$ values along the opposite edges of the cycle are unequal, that is, if $\Delta\Delta G_{iWT\rightarrow B1} \neq \Delta\Delta G_{iB2\rightarrow BB}$ and $\Delta\Delta G_{iWT\rightarrow B2} \neq \Delta\Delta G_{iB1\rightarrow BB}$, then the residues at the two positions are thermodynamically coupled either through a direct or indirect interaction (37). This is true in our DMCs for all PLs, either with or without κ-casein, indicating that the effects of B1 and B2 mutations are coupled in all cases.

The coupling energy is calculated as

$$\Delta\Delta G_{i\ (coupling)} = \Delta\Delta G_{iWT\rightarrow B2} - \Delta\Delta G_{iB1\rightarrow BB} = \Delta\Delta G_{iWT\rightarrow B1} - \Delta\Delta G_{iB2\rightarrow BB}.$$

RESULTS

Abolishing ATP hydrolysis significantly alters PL2 and PL3 but not PL1 dynamics

To study pore-loop dynamics by smFRET, we labeled ClpB molecules with two fluorescent dyes that comprise a FRET pair, Alexa Fluor 488 and Alexa Fluor 594, as in our preceding study (28). One dye was placed directly onto the pore loop of interest, and the second dye was located on a rigid reference position in the middle of the molecule (Fig. 1 *b* and *c*). We previously verified that labeling at these positions does not hinder the activity of ClpB (28). Based on this labeling scheme, it is expected that, as the flexible pore loop moves up and down along the axial channel, the relative distance between the two dyes would change, leading to fluctuations in the measured FRET efficiency signal. For smFRET measurements, we mixed the double-labeled ClpB protomers with a 100-fold molar excess of unlabeled ClpB protomers, using a refolding and reassembly procedure introduced previously (section "materials and methods"). This procedure yields ClpB hexamers that contain a single fluorescently labeled protomer. In case a functional mutation is present, we always combine the double-labeled ClpB mutant protomer with its corresponding unlabeled cysteine-less ClpB mutant protomers (see section "materials and methods") to ensure that the studied functional mutation is present in all protomers of the reassembled ClpB hexamer, rather than only in the fluorescently labeled protomer. To study the effect of abolished ATP hydrolysis on pore-loop dynamics, we first mutated a conserved glutamic acid residue, acting as an essential water-attacking base for ATP hydrolysis (7), within Walker B motifs in both NBDs (E271A/E668A, denoted as BB). Note that these mutations within the nucleotide-binding sites are distant from the pore loops, as can be seen from the structure of ClpB (Fig. 1). Homogeneous assembly and absence of ATPase activity in these mutants were verified by native PAGE chromatography and ATPase activity assays, respectively (Fig. S1). Pore-loop constructs with (BB) or without (wt) the mutations were analyzed by smFRET spectroscopy in aqueous solution in the presence of a saturating concentration of ATP (2 mM), either without or with the addition of the soluble model substrate protein κ-casein (38). We previously estimated that, with κ-casein, our results mostly represent the protein-bound protomers (28). Bursts of photons emitted as labeled ClpB molecules freely diffused through a focused laser beam were collected, and FRET efficiency histograms were constructed from the experimental data after selection of double-labeled molecules (see section "materials and methods"; Fig. 2).

In good agreement with our preceding study (28), the FRET efficiency histograms of the wt pore-loop constructs (without Walker B mutations) were broad, indicating dynamic heterogeneity, and displayed a shift to low FRET efficiency values upon κ-casein addition (Fig. S1). We previously verified that κ-casein addition does not affect the photophysical properties of our fluorescent dyes (as discussed in Supporting material, section "supplemental smFRET data analysis details"). We stress that FRET efficiency histograms in this study are used only for qualitative purposes. To quantitatively characterize the underlying pore-loop dynamics, we analyze fluorescent bursts on a photon-by-photon level, using H^2MM (27), a statistical tool for the analysis of single-molecule data, in which photon arrival times are used as input (section "materials and methods"). As previously (28), we found that these data are best described with two states, state 1 at low FRET efficiency and state 2 at high FRET efficiency, and used fixed FRET efficiency values for the analysis (as detailed in Supporting material, section "supplemental smFRET data analysis details" and Table S1). We assign the two FRET efficiency states to the up and down conformations of the pore loops along the axial channel, based on the H^2MM analysis and on previous structural triangulation calculations, as well as control measurements that verified the absence of relative motion of the NBDs themselves (28). It is possible that the up conformations of the pore loops serve to engage the substrate-protein and avoid its back-slipping, and the down conformations aid with the directional translocation of the substrate-protein across the axial channel. The H^2MM analysis was verified by stochastic recoloring of the data and segmentation analysis to confirm the two-state representation (Fig. S2) and by dwell time calculations, which were in agreement with the H^2MM-derived rates (Table S6). According to the H^2MM analysis, the transition rates between the two states were fast, $k_{12}^1 = 44{,}400 \pm 1800\ \mathrm{s}^{-1}$ and $k_{21}^1 = 20{,}600 \pm 1800\ \mathrm{s}^{-1}$

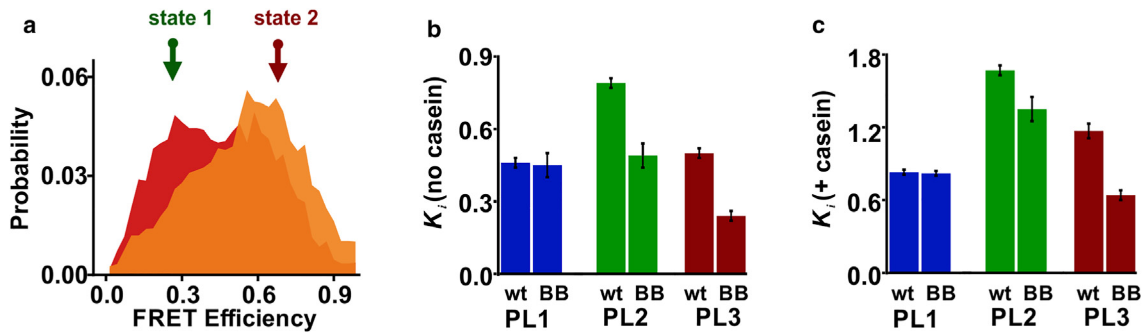

FIGURE 2 Effect of BB mutations on the dynamic equilibrium of PL1, PL2, and PL3. (*a*) FRET efficiency histograms of PL3 (*red*) and PL3 BB (*orange*), both in the presence of 25 μM κ-casein. Here and elsewhere below, single-molecule measurements were conducted using 1:100 labeled:unlabeled ClpB with 2 mM ATP. BB mutations are E271A/E668A (numbering as in the full-length TT ClpB). Arrows show the FRET efficiency values of two fixed states (same across different PL3 mutants) used in the H^2MM analysis of PL3 (Table S1). The positions of these two states were obtained from a global analysis of pore-loop data with and without κ-casein done previously (28) (detailed in Supporting material, under section "supplemental smFRET data analysis details"). See Fig. S1 for smFRET histograms of PL1, PL2, and PL3 wt and BB constructs with and without κ-casein. (*b*) H^2MM-derived equilibrium coefficients, K_i , for wt and BB mutants, measured without κ-casein. (*c*) H^2MM-derived equilibrium coefficients, K_i, for the same constructs, measured with 25 μM κ-casein. All K_i values are listed in Table S6. Overall higher values than in (*b*) are due to a clear increase in the population of the low-FRET state in all pore loops in the presence of κ-casein. In both (b) and (*c*), a differential effect of the BB mutation was registered (average values are reported, errors are SD, $n = 2$–3 repeats of the experiment). To see this figure in color, go online.

for PL1 without substrate-protein, and slightly slower upon κ-casein addition, $k_{12}^1 = 17{,}700 \pm 2200\ s^{-1}$ and $k_{21}^1 = 14{,}700 \pm 1700\ s^{-1}$ (errors from at least three repeats, see Table S6 for the transition rates of all other pore-loop types). These microsecond-timescale transition rates position pore-loop motions on a much faster timescale than the ATP hydrolysis in ClpB. To characterize the changes in the state populations of each pore-loop type (i), we used an effective equilibrium coefficient, $K_i = \frac{k_{21}^i}{k_{12}^i} = \frac{P_1^i}{P_2^i}$, defined as the population ratio of state 1 to that of state 2 (Table S6) (28). In response to κ-casein addition, the equilibrium coefficient of PL1, K_1, changed from 0.46 $\pm$ 0.02 to 0.83 $\pm$ 0.02, which was reflected in the shift to low-FRET values in the FRET efficiency histograms and fully agreed with our preceding results (28). PL2 and PL3 both also responded to κ-casein addition by a shift to low-FRET values (Fig. S1). Note that, according to our previous analysis (28), our measurements are consistent with pore-loop motions of around 10 Å (or two amino acids), representing significant structural fluctuations, which likely also involve adjacent structural elements.

In the BB constructs where ATP is bound and not hydrolyzing, all three PLs still displayed high transition rates between their two states, similar to the nonmutated constructs (Table S6). For example, PL1 BB without κ-casein showed $k_{12}^1 = 47{,}600 \pm 6000\ s^{-1}$ and $k_{21}^1 = 22{,}500 \pm 1900\ s^{-1}$, close to the values for PL1 wt. Furthermore, although the BB mutants still responded to κ-casein addition by a shift to low FRET efficiency values, their responses were modulated in a differential manner (Figs. 2 and S1). We quantified these κ-casein-induced shifts by using parameters derived from the H^2MM analysis (as summarized in Table S6). The results corroborate our previous study (28), in which we found that PL1 responds similarly to the substrate in the presence of either ATP or ADP, whereas PL2 and PL3 respond differently. Importantly, we did not observe this characteristic κ-casein-induced shift in the FRET efficiency histograms under conditions that favor ClpB disassembly, such as high salt and absence of added nucleotide (Fig. S3). Thus, the κ-casein-induced change in the pore-loop dynamics is a feature of ATP-bound and assembled ClpB complexes. Surprisingly, FRET efficiency histograms of PL3 BB displayed a shift to high FRET efficiency values compared to the corresponding histograms of the nonmutated samples, both with and without κ-casein (Figs. 2 *a* and S1). Furthermore, the derived H^2MM parameters for PL2 BB and PL3 BB mutants differed significantly from the results for their corresponding wt constructs, indicating a strong perturbation of the pore-loop dynamics by the BB mutations. These results are summarized in Figs. 2, and 6 *b* and *c*, Table S6. In particular, the effective equilibrium coefficients were decreased (Fig. 2; Table S6). This decrease was especially strong in the case of PL3, with K_3 changing from 0.50 $\pm$ 0.02 for PL3 wt to 0.24 $\pm$ 0.02 for PL3 BB, and from 1.17 $\pm$ 0.06 for PL3 with κ-casein to 0.64 $\pm$ 0.04 for PL3 BB with κ-casein. This change indicates that the high-FRET state of PL3, which corresponds to the up conformation, becomes more populated in the absence of ATP hydrolysis. Considering that the BB mutations are remote from the pore loops, this points to strong allosteric communication between the pore loops and the ATPase sites in ClpB. Therefore, even though pore loops fluctuate on the microsecond timescale, the dynamic equilibrium of PL2 and PL3 is significantly affected by the changes in the nucleotide state of the machine. In the PL1 samples, however, the difference between the wt and BB variants was absent, indicating that the dynamics of this pore loop are not affected by abolished ATP hydrolysis (28).

BB mutations perturb conformational fluctuations and allosteric paths of PL2 and PL3 but not of PL1

The effect of BB mutations on pore-loop motions can be rationalized in terms of the effect of mutations on conformational dynamics of ClpB and the pore loops and on the allosteric networks connecting the ATP-binding sites to the pore loops. To obtain information on dynamics and allosteric networks, we performed MD simulations of wt and BB variants of *E. coli* ClpB, for which high-resolution structures are available (9) (see section "materials and methods").

In equilibrium simulations, PCA provides significant insight into conformational fluctuations by highlighting the independent modes of motion that collectively determine the essential dynamics. The PCA approach relies on diagonalization of the covariance matrix of atomic fluctuations, which yields eigenvalues that characterize the amplitude of fluctuations and eigenvectors that correspond to the orthogonal directions with maximal variance (see section "materials and methods"). We focus on the eigenvectors corresponding to the top eigenvalues with the largest contribution to the variance. We perform this analysis on the pore loops of protomers 2–4, as both NBDs of these protomers are in an active (ATP-bound) state. For PL1 and PL2 of these protomers, both in the wild type and BB variant, the top 10 eigenvalues (principle components, PCs), arranged in decreasing order, contribute >80% of the variance; therefore, we restrict our analysis to the corresponding eigenvectors in all systems considered. Quantitative comparison between the essential subspaces comprising 10 eigenvectors of the wt or BB variants is made by using the root-mean-square inner product (RMSIP) between the two sets of eigenvectors (see section "materials and methods"). Intriguingly, pore loops display a distinct response to perturbation. PL1 loops demonstrate a high RMSIP value of ≅ 0.80, which reveals a strong similarity of motions of this set of pore loops and weak perturbation of their dynamics. Consistent with this observation, we note the large overlap between PC1 eigenvectors of PL1 loops in the BB variant and in the wt ClpB, ≅0.85. Swing-like motions of PL1 loops observed for the wt ClpB are largely preserved in the BB variant (Fig. 3 *a* and Video S1). A moderate overlap is found for PC2 eigenvectors of PL1 loops, ≅0.54, which emphasize torsional motions in both wt and BB variants (Fig. S4 *a*). Motions of PL2 and PL3 loops (Fig. 3 *b*, *c*, S4 *B* and *C*, Videos S2 and S3) are more strongly perturbed by the BB mutations than those of PL1 loops, with the RMSIP values of ≅0.64 for PL2 and 0.68 for PL3, respectively. Correspondingly, eigenvectors PC1 and PC2 of PL2 and PL3 loops in the BB variant have small to moderate overlap with the top two PCs in the wt ClpB, as indicated by cumulative overlaps of each of the top two PCs in the BB variant with respect to those in wt ClpB ranging between 0.09 and 0.51 (Table S2). Thus, although, for PL1, the two PCs of the BB variant corresponding to the top eigenvalues are well represented by the top two PCs of the wild-type ClpB, for PL2 and PL3, a larger set of PCs is required, corresponding to lower eigenvalues of the wild-type ClpB (Table S2).

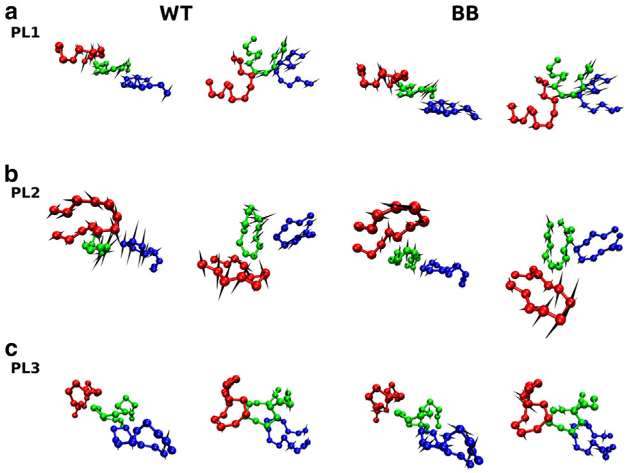

FIGURE 3 Effect of BB mutation on pore-loop dynamics. Motions associated with the PC1 eigenvector are shown for (*a*) PL1; (*b*) PL2, and (*c*) PL3 in protomers 2–4 (*blue, green, and red, respectively*) of the wild-type ClpB (*left panels*) and of the BB variant (*right panels*). Top and side views are shown. Directions of motions are indicated using spikes. Motions of PL1 loops are less affected by mutations than those of the hexamer and PL2-3 loops. See also Videos S1–S3. To see this figure in color, go online.

To reveal allosteric paths connecting Walker B and pore-loop sites within a ClpB subunit, we resorted to a graph theory-based approach (39–47). The network comprises nodes representing individual residues, located at C_α positions, connected by edges whose lengths are weighted by the residue-residue cross-correlations (see section "materials and methods"). It is important to note that the length of these path edges emphasizes the strength of allosteric coupling over the proximity in the Cartesian space of the residue pair. Thus, allosteric paths identified by this approach highlight the most effective propagation of allosteric coupling; therefore, we focused here on the optimal path, with the shortest length, and suboptimal paths, with slightly longer lengths (see section "materials and methods"). First, we determined up to 200 paths connecting the targeted mutation site from the Walker B regions of NBD1 ($NBD1_B$), E279, or of NBD2 ($NBD2_B$), E678, to the labeled residue of pore loop PL1 (A244), PL2 (A289) or PL3 (Y656). We examined three protomers from the high-resolution structure (9) and found that intra-protomer networks and allosteric paths are specific for each protomer. As shown in Tables S3 and S4, optimal paths identified in protomers of the wild-type ClpB are only slightly perturbed by the BB mutations. The limited changes to these paths are consistent with findings of previous studies, which noted that optimal paths are robust against perturbation (46). To obtain a broader understanding of the response of the allosteric network to these perturbations, and since current literature does not support

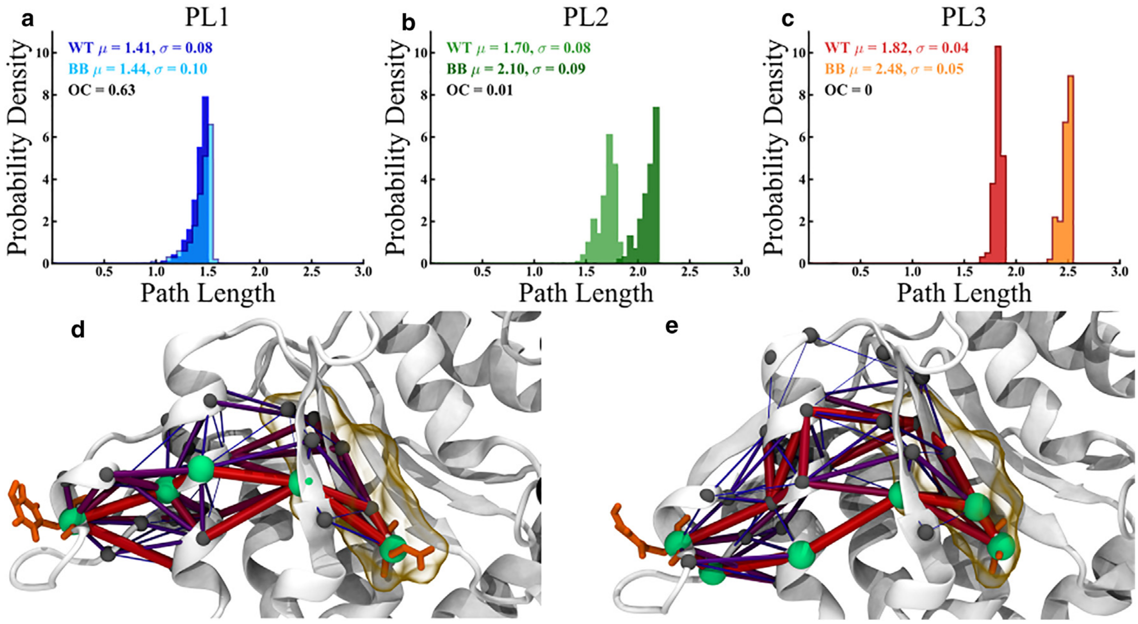

FIGURE 4 Computed allosteric paths connecting the Walker B and pore-loop regions in ClpB. Probability density distributions of the 200 shortest paths between Walker B residues targeted by mutations, E279 in NBD1 and E678 in NBD2, and the pore-loop residue labeled in FRET experiments, A244 in PL1, A289 in PL2, and Y656 in PL3 in one protomer of wt ClpB (*E. coli)* and BB mutant for (*a*) PL1 (*b*) PL2, and (*c*) PL3 wt and BB. The effect of perturbation on allosteric communication is weak in PL1 but strong in PL2 and PL3. Structural details of optimal and suboptimal paths are shown for PL3 (*d*) wt and (*e*) BB. Optimal paths (see Tables S3 and S4), which have the shortest length, illustrate the strongest set of allosteric couplings between nodes (*green*) of the allosteric network, which mediate the signaling between the Walker B site and the pore loop. The optimal path is slightly perturbed by BB mutations, whereas the ensemble of suboptimal paths (*purple*), which have longer path lengths, is strongly perturbed by mutations. Line thickness is proportional to the strength of the coupling. To see this figure in color, go online.

the occurrence of a single allosteric pathway (46), we therefore also studied the suboptimal paths. As shown in Figs. 4 and S5, the path length distributions of suboptimal paths have distinct behavior for the three pore loops. The PL1 distribution corresponds to stable allosteric coupling, with the similarity of path length distributions of wild-type and BB variants, quantified by the average overlap coefficient (OC) ∼0.44 ± 0.22 (see Fig. S5 *j* and section "materials and methods").

By contrast, the PL2 distributions indicate a strong perturbation in the allosteric coupling to the $NBD1_B$ region, with OC ∼0.16 ± 0.13 (Fig. S5 *j*). In the PL3 case, we found the largest shift in the strength of allosteric coupling compared with PL1 or PL2, with OC ∼0.09 ± 0.15 (Fig. S5 *j*). Thus, we find that BB mutants significantly altered the allosteric coupling of PL2 and PL3 dynamics, compared to PL1. The structural maps of allosteric paths (Figs. 4 and S6) illustrate the strong coupling of PL regions to active sites within the same NBD. The pattern of suboptimal paths that connect the Walker B region to PL1 remains largely unchanged in the BB mutant compared with the wild type (Fig. S6 *A* and *B*), whereas, in the case of PL2 (Fig. S6 *c* and *d*) and PL3 (Fig. S6 *e* and *f*), it is strongly altered. Overall, our computational analysis supports the experimental results, which indicate a weaker effect of the BB mutations on PL1 and a stronger effect on PL2 and PL3.

Absence of ATP hydrolysis strengthens κ-casein binding to pore loop 2

It was previously found that BB mutant of *E. coli* ClpB makes a stable interaction with model substrate proteins, for which it was referred to as a substrate trap (48). As noted above, in our smFRET experiments, we see a concentration-dependent κ-casein-induced shift to state 1 (low FRET), which indicates an increased population of state 1. We presume that, at intermediate κ-casein concentrations, there exist both bound and unbound ClpB. However, because thousands of such molecules are analyzed in the measurement, we obtain an effective concentration-dependent shift (Fig. S11) and can make use of this shift to estimate and compare κ-casein binding affinity to each pore loop by conducting smFRET κ-casein titrations.

To this end, we conducted smFRET measurements for PL1, PL2, and PL3 constructs as well as for their BB mutants in the presence of increasing concentrations of unlabeled κ-casein, and we carried out H^2MM analysis of these datasets with two fixed states, as described before. We calculated the ratios of H^2MM-derived populations of state 1 (at low FRET) with and without casein. Plots of these ratios against the concentrations of κ-casein yielded saturation curves for all three types of pore loops (Fig. 5). Fits of these curves to a simple binding isotherm revealed micromolar dissociation constants for PL1 and PL3 (2.9 and 0.9

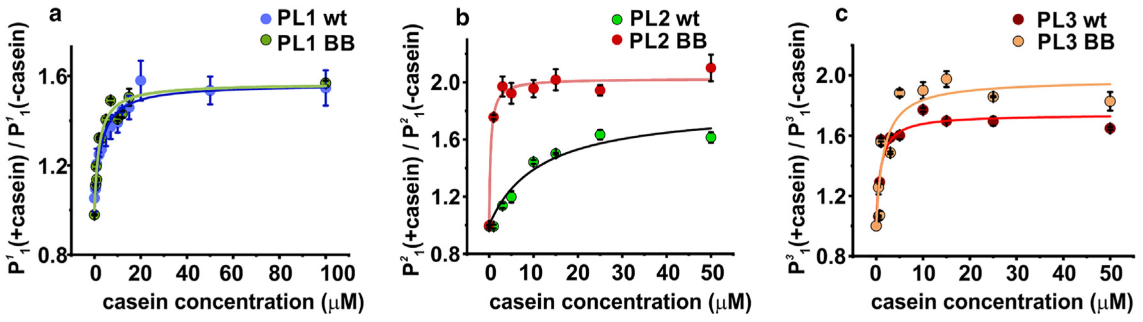

FIGURE 5 smFRET κ-casein titration experiments with wt and BB constructs. The average ratio of state 1 population with κ-casein to that without κ-casein is plotted (as circles) against casein concentration. Error is SD ($n = 3$) for PL1 and PL3 datasets, and SD ($n = 2$) for PL2 datasets. Solid lines are fits to a binding model (details in Table S5). To see this figure in color, go online.

μM, respectively; Table S5). In this analysis, neither PL1 nor PL3 showed dramatic differences in κ-casein binding between the nonmutated wt and the ATP-hydrolysis-deficient BB constructs. In contrast, however, there was a surprising 40-fold increase in the binding affinity for PL2 upon abolishing ATP hydrolysis, and the derived K_d changed from 11.3 μM in PL2 wt to 0.3 μM in PL2 BB construct. Based on this difference, it is plausible that the increased substrate binding to PL2 is involved in the substrate-trap effect noted in the past (48). In our data, in the high-affinity PL2 BB construct, the high-FRET state (state 2), corresponding to the down conformation of PL2, becomes more populated than in PL2 wt (0.67 $\pm$ 0.02 vs. 0.56 $\pm$ 0.01 in the wt; Table S6). Possibly, this conformation allows maximizing contacts with the substrate protein and this way stabilizes its binding and acts to prevent its back-slipping.

Both NBD1 and NBD2 affect pore-loop dynamics

In light of our results with the BB mutants, we set out to determine whether and how hydrolysis in NBD1 and NBD2 separately affects pore-loop dynamics. In particular, we wanted to find out if the nucleotide state of each NBD can affect only the pore loops that are located within that NBD or if this effect is longer-ranged.

To study the coupling of NBD1 and NBD2 to pore loops individually, we introduced single mutations into Walker B or Walker A motifs of either NBD. We generated single-NBD mutants which could not hydrolyze ATP in NBD1 (with mutation E271A, denoted as B1) or in NBD2 (E668A, B2). We also prepared mutants with abolished ATP binding to NBD1 (K204T, A1) or to NBD2 (K601A, A2). As a positive control for ATPase activity, we prepared a hyperactive mutant, which is expected to show elevated ATP hydrolysis rate due to an effect on its middle domain (K347A, hyper) (49). These constructs were homogeneously assembled, and their ATPase activity was found to be altered relative to the wt as expected, either lowered in the Walker mutants or increased in the hyperactive construct (Fig. S12). Having checked their bulk properties, we analyzed these constructs by smFRET spectroscopy.

All constructs displayed broad FRET efficiency histograms (Figs. 6, S13, and S14) with a shift to low FRET efficiency values upon κ-casein addition, and fast transition rates from the H^2MM analysis, on the same timescale as for the nonmutated pore-loop constructs (Table S6). However, the populations of the two states were affected, as indicated by the shifts in the FRET efficiency histograms as well as by the H^2MM-derived effective equilibrium coefficients, K_i, as is summarized in Fig. 6 *a–c*.

PL1 was least affected by the mutations in the NBDs, and both B1 and B2 mutations slightly increased the population of the low-FRET state (up conformation; Figs. 6, S13, and S14), even though this effect was surprisingly lost in the BB mutant (Fig. S1). The observation that this pore loop is affected by hydrolysis in either NBD indicates the presence of long-range allosteric communication in ClpB. PL2 was affected by all mutations in the same way: they all led to the increase in the population of the high-FRET (downward) conformation, which might aid with the substrate-protein binding. For example, abolishing ATP hydrolysis in NBD1 or in NBD2 caused K_2 to change from 0.79 $\pm$ 0.02 to 0.37 $\pm$ 0.01 (in PL2 B1 construct) or to 0.41 $\pm$ 0.01 (in PL2 B2), respectively (Table S6). Thus, the effect is more prominent when ATP is bound and not hydrolyzing in NBD1 than in NBD2, suggesting that there is stronger coupling of PL2 to NBD1 than NBD2. Nevertheless, the dynamic equilibrium of PL2 can be regulated by the nucleotide state of either NBD.

In PL3, the dynamics were strongly affected by the studied mutations, suggesting a significant mechano-chemical coupling of this pore loop to the NBDs. As already mentioned, in the absence of ATP hydrolysis in both NBDs (BB construct), the population of high-FRET (up) state was increased (Fig. 2). In contrast, B2, A2, and A1 mutations led to increase in the low-FRET state (downward conformation relative to wt), whereas B1 mutation had almost no effect (Figs. 6, S13, and S14). In the B2 construct, K_3 changed from 0.50 $\pm$ 0.02 in wt to 0.79 $\pm$ 0.03 in B2 mutant without κ-casein, and the difference was also present with κ-casein addition (1.17 $\pm$ 0.06 in wt and 1.51 $\pm$ 0.04 in the mutant; Table S6). According

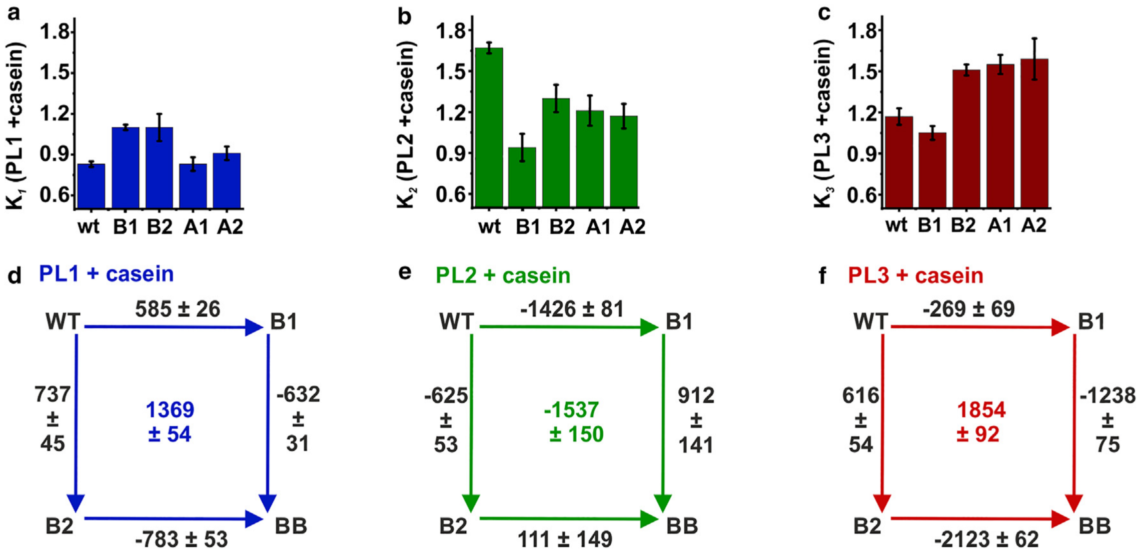

FIGURE 6 Effect of single NBD mutations on PL1, PL2, and PL3. (*a–c*) H^2MM-derived equilibrium coefficients, K_i, for sets of single Walker A/B mutants of PL1 (*a*), PL2 (*b*), and PL3 (*c*), all measured with 25 μM κ-casein (average values, errors are SD, $n = 2$–3 repeats). See Table S6 for K_i values without κ-casein and Figs. S13 and S14 for complete sets of smFRET histograms with single-NBD mutants. (*d–f*) DMCs for PL1, PL2, and PL3 with κ-casein (25 μM). The values along the edges are the average changes in free energy upon mutation, $\Delta\Delta G_i$ (in J.mol^{-1}), calculated from the H^2MM-derived K_i s detailed in section "materials and methods." The values at the centers of the squares are the average coupling energies, defined as the differences between free-energy changes of opposing edges (see section "materials and methods"). Error values are from the propagation of the SEs in K_i. The cycles without κ-casein are in Fig. S15. To see this figure in color, go online.

to these results, the dynamic equilibrium of PL3 can be shifted to either its up or down conformation depending on the nucleotide state of the NBDs. In the ATP-bound state in the absence of hydrolysis, PL3 favors a strained upward conformation. Once ATP hydrolysis occurs in the upper NBD (B2 mutant) or a nucleotide gets released from either the upper or the lower NBD (A1 and A2 mutants), this stabilization is lost and the low-FRET (downward) state becomes dominant. This population redistribution in PL3 is likely to be crucially involved in the substrate-protein translocation by ClpB.

DMCs expose significant coupling between NBDs

To further analyze the contributions of Walker B mutations in either NBD or in both, we employed DMCs (37). We calculated the free-energy differences for each construct, ΔG_i, by using the H^2MM-derived K_i values. We subsequently calculated the free-energy change upon each mutation, $\Delta\Delta G_i$, as the difference between the two corresponding ΔG_i values (detailed in section "materials and methods") and plotted thermodynamic cycles, where the resulting $\Delta\Delta G_i$ values are included along the edges (Figs. 6 *d—f* and S15). In these DMCs, the values along the opposite edges are not equal in all cases, indicating thermodynamic coupling (37). Considering that both the ATP-binding sites and the pore loops are separated in space and thus cannot interact directly, this result indicates long-range coupling. Interestingly, the coupling energies, calculated as the differences in $\Delta\Delta G_i$ between opposite edges (see section "materials and methods"), are positive for PL1 and PL3, both with and without κ-casein, suggesting that the introduction of one Walker mutation has a stabilizing effect on the other Walker mutation. The interaction energy for PL2, though, is negative.

DISCUSSION

The details of the translocation mechanism by AAA+ proteins are incompletely understood to date. Multiple experimental findings, as mentioned in the section "introduction," indicate that the functions of these molecular machines might involve complex conformational changes on multiple length- and timescales. Apart from the slow ATP-dependent sequential movements of protomers, as inferred from cryo-EM images, intra-protomer motions, such as fluctuations of pore loops, can also significantly contribute to the function of these proteins.

In this study, we selectively monitor the intra-protomer motions of individual pore loops along the axial channel of ClpB and how they are affected by nucleotide-binding site mutations. Consistent with our recently published experimental results (28) and with theoretical predictions (36), we find that the pore loops are moving on the microsecond timescale. Furthermore, we see that the ratio of the up and down conformations of the pore loops along the axial channel is modulated in response to the changes in the ATPase state of each of the NBDs, indicating that the pore loops and the NBDs are allosterically coupled. Interestingly, suppression of ATP hydrolysis in both

NBDs leads to a perturbation of PL2 and PL3, but not PL1, suggesting a different role for the latter. To characterize these couplings, we perform MD simulations of ClpB and calculate the allosteric paths that connect the NBDs to the pore loops. These simulations show clear perturbations of the allosteric network and of the pore-loop dynamics due to ATP-hydrolysis-abolishing mutations, in good agreement with our experimental findings. Surprisingly, each of the NBDs can affect all pore loops, even if they do not belong to the same NBD, suggesting a long-range allosteric regulation in ClpB. Indeed, by using our experimental equilibrium coefficients in a DMC analysis, where the cycles include wt, BB, and the single B1 and B2 mutants, with the mutations being present in all six protomers of the hexamer, we find that the effects of the single mutations are not independent, with a significant energy of interaction coupling them (Figs. 6 and S15) (37). Allosteric communication between the two nucleotide-binding sites, NBD1 and NBD2, in ClpB (50–52) and related proteins (53) could previously be inferred from functional studies of ATP hydrolysis. What we report here is, in contrast, an uncharacterized form of such a coupling, operating through the long-range effect on the pore loops and leading to a direct effect on the mechanism of protein translocation.

Our experiments demonstrate quite remarkably that the conformational dynamics of PL3 show differential response to mutations in either of the two NBDs (results are summarized in Fig. 7). This pore loop is highly conserved within the AAA+ family (5), suggesting that a similar NBD-pore-loop communication might exist in other AAA+ members. It is likely that the ATP-dependent change in the conformational equilibrium of PL3 facilitates the translocation of substrate proteins across the central pore of ClpB. These results are in agreement with the previously reported evidence for nucleotide-dependent pore-loop motions in ClpB (21) and ClpX (25,26) and are consistent with the captured large-scale (17 Å) longitudinal motions of the pore loops in a DNA-unwinding AAA+ hexamer (12). Thus, the up/down motions of the substrate-binding pore loops might be conserved in the AAA+ machines and possibly serve to pull the substrates across the hexameric rings. Our analysis shows that these up/down motions occur on the microsecond timescale but can be affected by the changes in the nucleotide state of the protein, which is modulated on the timescale of ATP binding and hydrolysis. Altered microsecond pore-loop motions, as well as their effects on average populations of the up and down states of the pore loops, may ultimately affect the dynamics of substrate translocation.

Thus, the coupling of pore loops to NBDs involves two timescales and is consistent with the Brownian-ratchet mechanism (54,55) for translocation by ClpB, as has been proposed in our preceding work (28,56). In this mechanism, the change in the population ratio of the pore loops

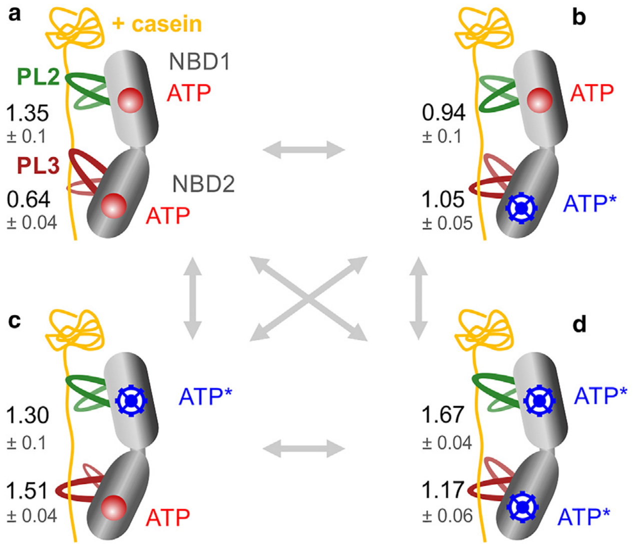

FIGURE 7 smFRET results suggest an ATP-dependent modulation of the pore-loop dynamics. (*a–d*) States of ClpB monomer are schematically shown, with bound *κ*-casein (in yellow) and pore loops PL2 and PL3 in green and dark red, respectively. PL1s are omitted for simplicity but included in Fig. S16. The ATPase states of the NBDs are depicted as follows: red circles, bound ATP molecules not undergoing hydrolysis (ATP arrested state); blue wheels, bound ATP undergoing hydrolysis, which corresponds to a mixture of ATP/ADP, although ATP is in excess in these measurements (2 mM). As a consequence of altered microsecond rates, PLs favor either an up or a down conformation depending on the ATPase state of the NBDs, and the size of the PLs on the scheme reflects their state occupancy from the H^2MM analysis. Numbers are the average H^2MM-derived equilibrium coefficients, K_i (errors are SD, $n = 2–3$). The conformational equilibrium of the pore loops periodically switches between the up/down states in response to the changing ATPase state of the machine, and this might facilitate substrate-protein translocation. To see this figure in color, go online.

that occurs upon ATP binding/hydrolysis corresponds to a shift between two free-energy surfaces, flat and structured, and results in a unidirectional, rectified substrate-protein translocation (55). Because of these two distinct timescales characterized, we can infer that the translocation by ClpB is likely to be faster than what can be expected based only on its ATP hydrolysis rate, in agreement with the recent study using optical tweezers (19). Indeed, since pore-loop motion is not fully coupled to substrate translocation, the microsecond motion of the former can readily lead to millisecond substrate translocation, as reported in that paper.

Our approach allows us to experimentally measure the substrate-binding affinities of distinct pore-loop types in ClpB. We find that pore loops 1 and 3 and their ATP-hydrolysis-deficient BB mutants bind to the substrate-protein *κ*-casein with similar affinities. In contrast, the ATP-hydrolysis-deficient PL2 BB mutant binds to the substrate ~40 times more strongly than the wild-type PL2. Our ability to measure substrate-protein affinities to distinct pore-loop types is entirely novel, but it lends support to previous findings that BB mutations result in stronger substrate-protein

binding to ClpB (48) and might offer a more nuanced explanation of these bulk observations. Considering that PL2 is the least conserved pore loop, its ATP-hydrolysis-dependent change in protein-binding affinity might act as a very effective filter for substrate-protein selection before they get passed to the lower NBD2 and bind to PL3. The latter is conserved in the AAA+ family (5), which might imply that substrate-protein recognition by PL3 is not highly specific. Indeed, ClpX, which contains a tyrosine pore loop analogous to PL3 of ClpB, was previously demonstrated to translocate a range of polypeptide substrates independently of the steric or chemical properties of their side chains (57). It might be that, if the bound substrate-protein passes the "filtration" by the less conserved PL2 within NBD1, it subsequently gets transferred into NBD2 and is committed to complete translocation across the axial pore of ClpB. Similarly, selectivity during substrate-protein recognition in ClpX is ensured by its RKH and pore-2 loops, which are poorly conserved in other AAA+ machines (25,58).

By summarizing our data in Fig. 7, we can speculate on how the pore loops in ClpB facilitate the ATP-dependent substrate-protein translocation. Given that our experiments are performed under saturating ATP concentrations (2 mM), that ATP binding is stochastic, and that ATP hydrolysis is orders of magnitude slower than pore-loop motions, all four states of ClpB that are depicted in Fig. 7 are likely to be present. From our results, it is plausible that ATP binding and hydrolysis events drive substrate-protein translocation by allosterically modulating the dynamic equilibrium of PL2 and PL3.

Although we have not directly measured substrate-protein translocation by ClpB, by considering the results from both our preceding (28) and current study, we can make assumptions on how ATP coordinates substrate-protein translocation. In particular, we can suggest that the changes in the dynamic equilibrium of PL3 are directly coupled to ATP-dependent protein translocation. Previously, we established that PL3 dynamics are correlated to bulk disaggregation activity of ClpB (28). Furthermore, we observed both previously (28) and currently that PL2 is ATP dependent but does not correlate to any bulk activities, neither disaggregation nor ATPase. We therefore assume that PL2 functions as a pawl, which acts to ensure unidirectional substrate-protein translocation. Regarding PL1, we previously found it to be nucleotide-type independent and do not observe effect on its dynamics upon abolishing ATP hydrolysis (in the BB construct). These results are consistent with the recent results from MD simulations (36). Although the dynamics of PL1 are independent of the machine's nucleotide state, we still expect this pore-loop type to be important for translocation, since it is likely to be essential for the initial substrate binding. Indeed, we also previously found that PL1 dynamics correlate with bulk disaggregation activity (28) and therefore expect PL1 to be crucial for the substrate-protein engagement and translocation, although we do not include it in Fig. 7 for simplicity. We include a summary of our results for all three PLs in Supporting material (Fig. S16) along with a further discussion of the potential translocation mechanism (Fig. S17).

Using Walker B mutants, we now find that PL2's affinity for substrate protein is dramatically increased when ATP is bound to both NBDs but not undergoing hydrolysis (BB construct or ATP arrested state; Fig. 7 *a*). This result indicates that simultaneous ATP binding to both NBDs, before ATP hydrolysis takes place, likely acts as a high-affinity state of ClpB that ensures substrate-protein binding and possibly prevents it from back-slipping.

At the same time, in this ATP arrested state (Fig. 7 *a*), PL3 is stabilized in an upward conformation, which might aid with the substrate-protein transfer into NBD2. PL3 binds the substrate protein, either to prepare for pulling or to act as a pawl. Either upon ATP hydrolysis in NBD2 (Fig. 7 *b*) or in both NBDs (Fig. 7 *d*), where PL3 samples the up/down states almost equally, it acts by pulling, whereas upon ATP hydrolysis in NBD1 (Fig. 7 *c*), where it favors the downward state, it likely acts as a pawl. Alternatively, the fast up and down transitions of PL3 (in Fig. 7 *b* and *d*) perhaps act to allow PL3 to reconfigure its binding to the substrate (this is similar to our findings with the enzyme adenylate kinase, which opens and closes multiple times to optimize substrate configuration (31)). ATP hydrolysis in NBD1 (Fig. 7 *c*) stabilizes the down conformation of PL3, likely promoting substrate pulling toward the exit of the channel. Note that the stabilization of the downward conformation, seen upon ATP hydrolysis in NBD1 (Fig. 7 *c*), also occurs upon ATP release from either NBD (Fig. S16).

ATP hydrolysis in NBD2, while ATP is bound to NBD1 (Fig. 7 *b*), allosterically modulates PL2, slightly stabilizing its downward conformation. This action likely allows PL3 to pull or reconfigure, whereas PL2 acts as a pawl. As mentioned above, ATP binding to NBD2, while NBD1 is undergoing ATP hydrolysis (Fig. 7 *c*), allosterically modulates PL3, stabilizing its downward conformation. This action likely allows PL2 to release its contact from the substrate and allows PL3 to act as a pawl or to pull on the substrate protein.

CONCLUSIONS

To conclude, our results establish a considerably long-ranged allosteric communication between the ATP-binding sites of ClpB and its pore loops, which are responsible for substrate translocation, thus establishing the pore loops as active participants during ATP-dependent translocation. Importantly, although previous studies have indicated that pore loops bind to substrate proteins and possibly undergo

conformational changes, this is the first direct and detailed biophysical characterization of the ATP-dependent regulation of the real-time dynamics of three distinct types of pore loops by each of the two ATP-binding sites. Remarkably, we find a significant interaction between the two ATP-binding sites in their effect on pore loops. This coupling modulates the dynamic equilibrium between the rapidly exchanging up/down states of the pore loops, thereby facilitating the ATP-dependent unidirectional translocation of protein substrates. Our smFRET measurements, coupled with MD simulations and mutational analysis, allow us to obtain a comprehensive picture of these allosteric interactions and to propose how dynamics on the slow timescale of ATP hydrolysis affects the much faster motions of protein-translocating structural elements. These results, and the methods presented here, should be relevant to the studies of multiple additional AAA+ proteins.

DATA AND CODE AVAILABILITY

Data generated in this work are available upon reasonable request.

SUPPORTING MATERIAL

Supporting material can be found online at https://doi.org/10.1016/j.bpj.2024.01.002.

AUTHOR CONTRIBUTIONS

M.I. designed and performed research, analyzed data, and wrote the paper. H.M. designed and performed research and contributed analytic tools. A.D., Z.Z., and G.S. designed research, contributed analytic tools, and analyzed data. I.R. and G.H. designed research and wrote the paper.

ACKNOWLEDGMENTS

We thank Prof. Amnon Horovitz for the advice on the DMC analysis. M.I. was the recipient of an EMBO Long-Term Fellowship (ALTF 317–2018) and the IASH Fellowship for International Postdoctoral Fellows. H.M. was supported by the Planning and Budgeting Committee of the Council for Higher Education of Israel. G.H. was funded by the European Research Council under the European Union's Horizon 2020 research and innovation programme (grant agreement no. 742637), by NSF-BSF grant no. 2021700, and by the Grand Center for Sensors and Security. G.H. is the incumbent of the Hilda Pomeraniec Memorial Professorial Chair. G.S. was funded by NSF-BSF grant MCB-2136816. This work used Bridges-2 resources at the Pittsburgh Supercomputer Center through allocation MCB170020 to G.S. from the Advanced Cyberinfrastructure Coordination Ecosystem: Services & Support (ACCESS) program, which is supported by National Science Foundation grants #2138259, #2138286, #2138307, #2137603, and #2138296.

DECLARATION OF INTERESTS

The authors declare no competing interests.

SUPPORTING CITATIONS

References (59–69) appear in the supporting material.

REFERENCES

1. Jessop, M., J. Felix, and I. Gutsche. 2021. AAA+ ATPases: structural insertions under the magnifying glass. *Curr. Opin. Struct. Biol.* 66:119–128.
2. Gates, S. N., and A. Martin. 2020. Stairway to translocation: AAA+ motor structures reveal the mechanisms of ATP-dependent substrate translocation. *Protein Sci.* 29:407–419.
3. Crampton, D. J., S. Mukherjee, and C. C. Richardson. 2006. DNA-induced switch from independent to sequential dTTP hydrolysis in the bacteriophage T7 DNA helicase. *Mol. Cell.* 21:165–174.
4. Enemark, E. J., and L. Joshua-Tor. 2006. Mechanism of DNA translocation in a replicative hexameric helicase. *Nature.* 442:270–275.
5. Neuwald, A. F., L. Aravind, …, E. V. Koonin. 1999. AAA+: A class of chaperone-like ATPases associated with the assembly, operation, and disassembly of protein complexes. *Genome Res.* 9:27–43.
6. Weibezahn, J., P. Tessarz, …, B. Bukau. 2004. Thermotolerance requires refolding of aggregated proteins by substrate translocation through the central pore of ClpB. *Cell.* 119:653–665.
7. Hanson, P. I., and S. W. Whiteheart. 2005. AAA+ proteins: have engine, will work. *Nat. Rev. Mol. Cell Biol.* 6:519–529.
8. Lee, S., M. E. Sowa, …, F. T. F. Tsai. 2003. The structure of ClpB: a molecular chaperone that rescues proteins from an aggregated state. *Cell.* 115:229–240.
9. Rizo, A. N., J. Lin, …, D. R. Southworth. 2019. Structural basis for substrate gripping and translocation by the ClpB AAA+ disaggregase. *Nat. Commun.* 10:2393.
10. Lee, J., N. Sung, …, F. T. F. Tsai. 2017. Structural determinants for protein unfolding and translocation by the Hsp104 protein disaggregase. *Biosci. Rep.* 37.
11. Zhang, S., S. Zou, …, Y. Mao. 2022. USP14-regulated allostery of the human proteasome by time-resolved cryo-EM. *Nature.* 605:567–574.
12. Gai, D., R. Zhao, …, X. S. Chen. 2004. Mechanisms of conformational change for a replicative hexameric helicase of SV40 large tumor antigen. *Cell.* 119:47–60.
13. Ripstein, Z. A., S. Vahidi, …, L. E. Kay. 2020. A processive rotary mechanism couples substrate unfolding and proteolysis in the ClpXP degradation machinery. *Elife.* 9, e52158.
14. Fei, X., T. A. Bell, …, R. T. Sauer. 2020. Structures of the ATP-fueled ClpXP proteolytic machine bound to protein substrate. *Elife.* 9, e52774.
15. Tsai, F. T., and C. P. Hill. 2020. Same structure, different mechanisms? *Elife.* 9, e56501.
16. Martin, A., T. A. Baker, and R. T. Sauer. 2005. Rebuilt AAA + motors reveal operating principles for ATP-fuelled machines. *Nature.* 437:1115–1120.
17. Baytshtok, V., J. Chen, …, R. T. Sauer. 2017. Covalently linked HslU hexamers support a probabilistic mechanism that links ATP hydrolysis to protein unfolding and translocation. *J. Biol. Chem.* 292:5695–5704.
18. Aubin-Tam, M. E., A. O. Olivares, …, M. J. Lang. 2011. Single-molecule protein unfolding and translocation by an ATP-fueled proteolytic machine. *Cell.* 145:257–267.
19. Avellaneda, M. J., K. B. Franke, …, S. J. Tans. 2020. Processive extrusion of polypeptide loops by a Hsp100 disaggregase. *Nature.* 578:317–320.
20. Cho, C., J. Jang, …, J. J. Song. 2019. Structural basis of nucleosome assembly by the Abo1 AAA+ ATPase histone chaperone. *Nat. Commun.* 10:5764.
21. Schlieker, C., J. Weibezahn, …, A. Mogk. 2004. Substrate recognition by the AAA+ chaperone ClpB. *Nat. Struct. Mol. Biol.* 11:607–615.

22. Hinnerwisch, J., W. A. Fenton, …, A. L. Horwich. 2005. Loops in the central channel of ClpA chaperone mediate protein binding, unfolding, and translocation. *Cell.* 121:1029–1041.

23. Siddiqui, S. M., R. T. Sauer, and T. A. Baker. 2004. Role of the processing pore of the ClpX AAA+ ATPase in the recognition and engagement of specific protein substrates. *Genes Dev.* 18:369–374.

24. Deville, C., M. Carroni, …, H. R. Saibil. 2017. Structural pathway of regulated substrate transfer and threading through an Hsp100 disaggregase. *Sci. Adv.* 3, e1701726.

25. Martin, A., T. A. Baker, and R. T. Sauer. 2008. Diverse pore loops of the AAA+ ClpX machine mediate unassisted and adaptor-dependent recognition of ssrA-tagged substrates. *Mol. Cell.* 29:441–450.

26. Martin, A., T. A. Baker, and R. T. Sauer. 2008. Pore loops of the AAA+ ClpX machine grip substrates to drive translocation and unfolding. *Nat. Struct. Mol. Biol.* 15:1147–1151.

27. Pirchi, M., R. Tsukanov, …, E. Nir. 2016. Photon-by-Photon Hidden Markov Model Analysis for Microsecond Single-Molecule FRET Kinetics. *J. Phys. Chem. B.* 120:13065–13075.

28. Mazal, H., M. Iljina, …, G. Haran. 2021. Ultrafast pore-loop dynamics in a AAA+ machine point to a Brownian-ratchet mechanism for protein translocation. *Sci. Adv.* 7, eabg4674.

29. Mazal, H., M. Iljina, …, G. Haran. 2019. Tunable microsecond dynamics of an allosteric switch regulate the activity of a AAA+ disaggregation machine. *Nat. Commun.* 10:1438.

30. Nørby, J. G. 1988. Coupled assay of Na+,K+-ATPase activity. *Methods Enzymol.* 156:116–119.

31. Aviram, H. Y., M. Pirchi, …, G. Haran. 2018. Direct observation of ultrafast large-scale dynamics of an enzyme under turnover conditions. *Proc. Natl. Acad. Sci. USA.* 115:3243–3248.

32. Kapanidis, A. N., N. K. Lee, …, S. Weiss. 2004. Fluorescence-aided molecule sorting: analysis of structure and interactions by alternating-laser excitation of single molecules. *Proc. Natl. Acad. Sci. USA.* 101:8936–8941.

33. Grant, B. J., A. P. C. Rodrigues, …, L. S. D. Caves. 2006. Bio3d: an R package for the comparative analysis of protein structures. *Bioinformatics.* 22:2695–2696.

34. Eswar, N., D. Eramian, …, A. Sali. 2008. Protein structure modeling with MODELLER. *Methods Mol. Biol.* 426:145–159.

35. Malde, A. K., L. Zuo, …, A. E. Mark. 2011. An Automated Force Field Topology Builder (ATB) and Repository: Version 1.0. *J. Chem. Theor. Comput.* 7:4026–4037.

36. Damre, M., A. Dayananda, …, R. I. Dima. 2021. Factors underlying asymmetric pore dynamics of disaggregase and microtubule-severing AAA+ machines. *Biophys. J.* 120:3437–3454.

37. Horovitz, A. 1996. Double-mutant cycles: a powerful tool for analyzing protein structure and function. *Folding Des.* 1:R121–R126.

38. Woo, K. M., K. I. Kim, …, C. H. Chung. 1992. The heat-shock protein ClpB in Escherichia coli is a protein-activated ATPase. *J. Biol. Chem.* 267:20429–20434.

39. Atilgan, A. R., P. Akan, and C. Baysal. 2004. Small-world communication of residues and significance for protein dynamics. *Biophys. J.* 86:85–91.

40. Chennubhotla, C., and I. Bahar. 2006. Markov propagation of allosteric effects in biomolecular systems: application to GroEL-GroES. *Mol. Syst. Biol.* 2:36.

41. Sethi, A., J. Eargle, …, Z. Luthey-Schulten. 2009. Dynamical networks in tRNA:protein complexes. *Proc. Natl. Acad. Sci. USA.* 106:6620–6625.

42. Rivalta, I., M. M. Sultan, …, V. S. Batista. 2012. Allosteric pathways in imidazole glycerol phosphate synthase. *Proc. Natl. Acad. Sci. USA.* 109:E1428–E1436.

43. Miao, Y., S. E. Nichols, …, J. A. McCammon. 2013. Activation and dynamic network of the M2 muscarinic receptor. *Proc. Natl. Acad. Sci. USA.* 110:10982–10987.

44. Gasper, P. M., B. Fuglestad, …, J. A. McCammon. 2012. Allosteric networks in thrombin distinguish procoagulant vs. anticoagulant activities. *Proc. Natl. Acad. Sci. USA.* 109:21216–21222.

45. Scarabelli, G., and B. J. Grant. 2014. Kinesin-5 allosteric inhibitors uncouple the dynamics of nucleotide, microtubule, and neck-linker binding sites. *Biophys. J.* 107:2204–2213.

46. Van Wart, A. T., J. Durrant, …, R. E. Amaro. 2014. Weighted Implementation of Suboptimal Paths (WISP): An Optimized Algorithm and Tool for Dynamical Network Analysis. *J. Chem. Theor. Comput.* 10:511–517.

47. Verkhivker, G. M., and L. Di Paola. 2021. Dynamic Network Modeling of Allosteric Interactions and Communication Pathways in the SARS-CoV-2 Spike Trimer Mutants: Differential Modulation of Conformational Landscapes and Signal Transmission via Cascades of Regulatory Switches. *J. Phys. Chem. B.* 125:850–873.

48. Weibezahn, J., C. Schlieker, …, A. Mogk. 2003. Characterization of a trap mutant of the AAA+ chaperone ClpB. *J. Biol. Chem.* 278:32608–32617.

49. Lipińska, N., S. Ziętkiewicz, …, K. Liberek. 2013. Disruption of ionic interactions between the nucleotide binding domain 1 (NBD1) and middle (M) domain in Hsp100 disaggregase unleashes toxic hyperactivity and partial independence from Hsp70. *J. Biol. Chem.* 288:2857–2869.

50. Schlee, S., Y. Groemping, …, J. Reinstein. 2001. The chaperone function of ClpB from Thermus thermophilus depends on allosteric interactions of its two ATP-binding sites. *J. Mol. Biol.* 306:889–899.

51. Mogk, A., C. Schlieker, …, B. Bukau. 2003. Roles of individual domains and conserved motifs of the AAA+ chaperone ClpB in oligomerization, ATP hydrolysis, and chaperone activity. *J. Biol. Chem.* 278:17615–17624.

52. Watanabe, Y. H., K. Motohashi, and M. Yoshida. 2002. Roles of the two ATP binding sites of ClpB from Thermus thermophilus. *J. Biol. Chem.* 277:5804–5809.

53. Hattendorf, D. A., and S. L. Lindquist. 2002. Cooperative kinetics of both Hsp104 ATPase domains and interdomain communication revealed by AAA sensor-1 mutants. *EMBO J.* 21:12–21.

54. Astumian, R. D., S. Mukherjee, and A. Warshel. 2016. The Physics and Physical Chemistry of Molecular Machines. *ChemPhysChem.* 17:1719–1741.

55. Hwang, W., and M. Karplus. 2019. Structural basis for power stroke vs. Brownian ratchet mechanisms of motor proteins. *Proc. Natl. Acad. Sci. USA.* 116:19777–19785.

56. Riven, I., H. Mazal, …, G. Haran. 2023. Fast dynamics shape the function of the AAA+ machine ClpB: lessons from single-molecule FRET spectroscopy. *FEBS J.* 290:3496–3511.

57. Barkow, S. R., I. Levchenko, …, R. T. Sauer. 2009. Polypeptide translocation by the AAA+ ClpXP protease machine. *Chem. Biol.* 16:605–612.

58. Fei, X., T. A. Bell, …, R. T. Sauer. 2020. Structural basis of ClpXP recognition and unfolding of ssrA-tagged substrates. *Elife.* 9, e61496.

59. Iljina, M., H. Mazal, …, G. Haran. 2021. Entropic Inhibition: How the Activity of a AAA+ Machine Is Modulated by Its Substrate-Binding Domain. *ACS Chem. Biol.* 16:775–785.

60. Kamberaj, H., and A. van der Vaart. 2009. Correlated motions and interactions at the onset of the DNA-induced partial unfolding of Ets-1. *Biophys. J.* 96:1307–1317.

61. McGibbon, R. T., K. A. Beauchamp, …, V. S. Pande. 2015. MDTraj: A Modern Open Library for the Analysis of Molecular Dynamics Trajectories. *Biophys. J.* 109:1528–1532.

62. Harris, C. R., K. J. Millman, …, T. E. Oliphant. 2020. Array programming with NumPy. *Nature.* 585:357–362.

63. Tama, F., and Y. H. Sanejouand. 2001. Conformational change of proteins arising from normal mode calculations. *Protein Eng.* 14:1–6.

64. Yang, L., G. Song, …, R. L. Jernigan. 2008. Close correspondence between the motions from principal component analysis of multiple

HIV-1 protease structures and elastic network modes. *Structure.* 16:321–330.

65. Amadei, A., B. L. de Groot, …, H. J. Berendsen. 1999. A kinetic model for the internal motions of proteins: diffusion between multiple harmonic wells. *Proteins.* 35:283–292.

66. Girvan, M., and M. E. J. Newman. 2002. Community structure in social and biological networks. *Proc. Natl. Acad. Sci. USA.* 99:7821–7826.

67. Bradley, E. L. 1985. Overlapping Coefficient. *In* Encyclopedia of Statistical Sciences. John Wiley.

68. Weitzman, M. S. 1970. Measures of Overlap of Income Distributions of White and Negro Families in the United States. US Bureau of the Census.

69. Deville, C., K. Franke, …, H. R. Saibil. 2019. Two-Step Activation Mechanism of the ClpB Disaggregase for Sequential Substrate Threading by the Main ATPase Motor. *Cell Rep.* 27:3433–3446.e4.

Biophysical Journal, Volume *123*

Supplemental information

Single-molecule FRET probes allosteric effects on protein-translocating pore loops of a AAA+ machine

Marija Iljina, Hisham Mazal, Ashan Dayananda, Zhaocheng Zhang, George Stan, Inbal Riven, and Gilad Haran

Supplemental Information for

Single-molecule FRET probes the allosteric effect of ATP on the protein-translocating pore loops of a AAA+ machine

Marija Iljina[1], Hisham Mazal[1,2], Ashan Dayananda[3], Zhaocheng Zhang[3], George Stan[3], Inbal Riven[1] and Gilad Haran[1]

[1]Department of Chemical and Biological Physics, Weizmann Institute of Science, Rehovot 761001, Israel.

[2]Present Address: Max Planck Institute for Science of Light, Staudtstrasse 2, 90158 Erlangen, Germany.

[3]Department of Chemistry, University of Cincinnati, Cincinnati, OH 45221, United States.

Correspondence and requests for materials should be addressed to G.H. (email:gilad.haran@weizmann.ac.il)

Contents

Supplemental smFRET Data Analysis Details 3
Fixed states for H^2MM analysis 3
H^2MM analysis validations 3
κ-casein addition does not impact the photophysical properties of Alexa Fluor dyes 4
Supplemental Computational Methods: 4
Molecular dynamics simulations 4
Dynamic Cross-Correlation Matrix 4
Principal component analysis 5
Optimal and suboptimal path analysis 6
Supplemental Figures: 7
Figure S1. Double-Walker B (BB) mutants of ClpB show correct assembly but abolished ATP hydrolysis rate and display differential behavior in smFRET experiments. 7
Figure S2. H^2MM analysis of Walker BB mutants. 8
Figure S3. FRET efficiency histograms from experiments under assembly-disfavoring conditions. 9
Figure S4. Pore loop motions associated with PC2 eigenvectors.......... 10
Figure S5. Path length distributions in allosteric signaling in ClpB configurations. 11
Figure S6. Optimal and suboptimal paths connecting the Walker B regions and pore loops within a ClpB protomer. 12
Figure S7. Equilibration of MD trajectories.......... 13
Figure S8. Nucleotide stability within the binding site.......... 14
Figure S9. Interaction between nucleotide and ClpB. 15
Figure S10. DCCM convergence. 16
Figure S11. Representative FRET efficiency histograms from single-molecule κ-casein titration assay. 17
Figure S12. Single mutants of ClpB display correct assembly but modified ATPase activity. 18
Figure S13. FRET efficiency histograms of NBD1 mutants. 19
Figure S14. FRET efficiency histograms of NBD2 mutants. 20
Figure S15. Double-mutant cycles 21
Figure S16. Summary of all smFRET results suggests an ATP-dependent modulation of pore-loop dynamics.......... 22
Figure S17. Examples of potential routes leading to substrate-protein translocation by ClpB. 23
Supplemental Movies: 24
Movie SM1.......... 24
Movie SM2.......... 24

Movie SM3 24
Supplemental Data Summary Tables: 25
Table S1. FRET efficiency values of the two pore loop states as obtained from the global H^2MM analysis of wild-type pore-loop constructs, measured with or without κ-casein 25
Table S2. Cumulative overlap of the top 2 principal components of pore loops in the BB variant over the top 10 principal components of the corresponding pore loops in wild-type ClpB. 25
Table S3: Optimal paths between Walker B sites and pore loops in NBD1, PL1 and PL2, in wild type ClpB and BB mutants, derived from the MD simulations of *E. coli* ClpB. 26
Table S4: Optimal paths between Walker B sites and pore loops in NBD2, PL3, in wild type ClpB and BB mutants, derived from the MD simulations of *E. coli* ClpB. 26
Table S5. Substrate κ-casein binding constants to individual pore-loop mutants, derived from H^2MM analysis of single-molecule κ-casein titration measurements. 26
Tables S6: H^2MM Analysis Outputs. 27
PL1: WT and double mutants. 27
PL2: WT and double mutants. 27
PL3: WT and double mutants. 27
PL1: Single-NBD mutants 28
PL1: Other conditions 28
PL2: Single-NBD mutants 29
PL2: Other conditions 29
PL3: Single-NBD mutants 30
PL3: Other conditions 30
Supplemental References 31

Supplemental smFRET Data Analysis Details:

Fixed states for H^2MM analysis

As in our previous paper (1), here we characterized pore-loop dynamics using the H^2MM algorithm, a photon-by-photon hidden Markov modeling approach capable of quantifying microsecond-timescale dynamics in single-molecule FRET data (2). We previously determined two discrete FRET efficiency states to be essential and sufficient for the modeling of pore-loop dynamics. This was done by first using a model with 9-10 equally spaced FRET efficiency states, and finding that the derived free energy profiles showed only two major minima, which we assigned to 'up' and 'down' conformations of the pore loops (1). Therefore, here we also used the two-state H^2MM model for all data analysis presented in this study. Furthermore, we previously globally analyzed wild-type pore-loop constructs measured with and without a saturating concentration of κ-casein (20 μM), assuming that the two FRET efficiency states for each pore loop remain the same under both conditions (1). In the present work, the FRET efficiency values for each pore loop from this global analysis (listed in Table S1) are used as fixed inputs for the modelling of the datasets of all generated mutants of the corresponding pore-loop construct. This is done in order to simplify our analysis and to ensure the most direct comparison between the state population redistributions as a consequence of introduced mutations.

H^2MM analysis validations

This H^2MM analysis was validated through recoloring and segmentation analyses, which were described in detail in our preceding study (Fig. S5 in Mazal *et al.* (1)). Briefly, in the statistical recoloring analysis, described previously (1,3), the arrival times of photons, detected in the smFRET experiment, are kept unchanged, whereas the identity ('color') of photons, i.e., whether they result from the emission by AF488 or AF595 dye, is erased. The photons are subsequently 'recolored' using a Monte-Carlo simulation based on the model parameters derived from the H^2MM. Average FRET efficiencies for every burst are calculated, and a histogram is generated and compared to the experimentally measured histogram. Agreement between the two histograms indicates valid H^2MM modeling (Fig. S2). For the segmentation analysis, previously described (1), the most probable sequence of states in each burst is used, as obtained from the H^2MM analysis and the Viterbi algorithm (which is an algorithm that delineates the single best state sequence in each trajectory). Based on the assignment of each segment within the burst to state 1 or state 2, FRET efficiency histograms are calculated. It is expected that the calculated histograms will show a clear separation into two states for a valid two-state model (Fig. S2). We further validated the two-state analysis by calculating dwell time distributions, following a previously described procedure using a custom-written likelihood-weighted segmentation algorithm (3). This procedure analyzes the distribution of dwell times that are spent by the pore loops at each of their two states. For any photon trajectory, this analysis does not only take into account the most probable sequence of states (classically obtained by the Viterbi algorithm). Rather, every possible sequence of states contributes to the dwell time a fraction of a count that is equal to the likelihood of this sequence. Integrated dwell time distributions are computed and fitted to single exponential functions to derive the transition rates. Agreement between transition rates, derived from H^2MM, with the rates from the dwell time analysis provides the strongest indication for valid modeling (Tables S6).

κ-casein addition does not impact the photophysical properties of Alexa Fluor dyes

In our previous smFRET studies of ClpB, we verified that κ-casein addition has no effect on the photophysical properties of Alexa Fluor dyes. We reported that the presence of 25 μM of unlabeled κ-casein in our measurements has no effect on the mean numbers of photons per burst, mean photon rates or average burst lengths (as detailed before (4)). Furthermore, we observed identical FRET efficiency histograms in the presence and in the absence of κ-casein for two separate ClpB constructs with Alexa Fluor dyes located on rigid positions within NBD1 and NBD2 (1,4).

Supplemental Computational Methods:

Molecular dynamics simulations

Wild-type ClpB simulations involve solvation of the ATPase in a cubic box with dimensions $\sim 170 \times 170 \times 170$ Å^3, with 138913 water molecules represented using the single point charge (SPC) model and neutralized by adding 105 Na ions. The simulation box includes a total of 452304 atoms. Periodic boundary conditions (PBC) were applied in the three dimensions. For ClpB mutants, the protein structure was solvated in a dodecahedral box with dimensions $\sim 115 \times 163 \times 163$ Å^3 with 85780 water molecules, represented using the single point charge (SPC) model. 93 Na ions were added to the system to neutralize charges, resulting in a system with 292845 atoms. Energy minimization of the solvated systems was performed using the steepest descent algorithm for 50000 steps with convergence achieved when the maximum force reached a value smaller than 1000 kJ/ (mol · nm). Next, two equilibration steps were performed. First, NVT simulations were performed for 500 ps using the leapfrog integrator, with T = 300 K, and harmonic restraints applied to heavy atoms of the protein with a spring constant of 1000 kJ/ (mol · nm^2). In the second equilibration step, NPT simulations of the restrained system were performed for 500 ps with the pressure maintained at the constant value of 1 atm using the Parrinello Rahman algorithm (5). The time step in all molecular dynamics (MD) simulations was 2 fs. After removal of restraints, five unbiased NPT simulations of trajectories were performed for 150 ns (wild-type) and 150 ns (BB mutant). Thus, wild-type simulations, including previous data (6), comprised 150 ns for each MD trajectory. For analysis purposes, the first 10 ns of each trajectory were not included, and data frames were saved every 100 ps. Equilibration of simulation trajectories is assessed using root-mean-square deviations (Fig. S7). The stability of nucleotide location is evaluated by determining the distance between the center of mass of the nucleotide and the C_α atom of the Walker B mutation position within the same nucleotide binding site (Fig. S8), and the stability of nucleotide-ClpB interactions is quantified by calculating the total interaction energy, comprising Coulomb and Lennard-Jones terms for atom pairs within the cutoff distance of 12 Å, between each nucleotide and the ClpB hexamer (Fig. S9).

Dynamic Cross-Correlation Matrix

We used the Bio3D package (7) to determine the Dynamic Cross Correlation Matrix (DCCM) of position fluctuations of C_α atoms of protein residues, which quantifies the time-dependent residue-residue directional correlations. DCCM is an N × N matrix, where N is the number of residues, where each element C_{ij} corresponds to the dynamic cross-correlation between residues i and j:

$$C_{ij}(t) = \langle \Delta \mathbf{r}_i(t') \cdot \Delta \mathbf{r}_j(t') \rangle / (\langle \|\Delta \mathbf{r}_i(t')\|^2 \rangle \langle \|\Delta \mathbf{r}_j(t')\|^2 \rangle)^{1/2}$$

Here, $\triangle \mathbf{r}_i (t') = \mathbf{r}_i (t') - \langle \mathbf{r}_i \rangle$ denotes the instantaneous position fluctuation of residue i from its mean. Ensemble averages over all time frames up to time t and all trajectories are indicated by $\langle\cdot\rangle$. C_{ij} values range from -1 to 1, with motions of i and j atoms in the same direction corresponding to positive C_{ij} values, and motions in opposite directions to negative values. Convergence of the DCCM matrix was assessed using the mean square distance R(t) between correlation values of residue pairs at successive times, $R(t) = (1/N_p) \sum_{(ij)} (C_{ij} (t) - C_{ij} (t - \tau))^2$, where N_p is the number of residue pairs and the time interval $\tau = 10$ ns(5). Here, C_{ij} is evaluated using data frames up to the total simulation time per trajectory $t \leqslant 150$ ns. As shown in Figure S10, DCCM convergence for wild type and BB simulations is achieved within approximately 80 ns.

Principal component analysis

To capture important modes of pore loop dynamics, we performed principal component analysis (PCA) applied to the C_α positional fluctuations of the wild-type ClpB and the BB variant. In separate calculations, we performed PCA for each of the PL1, PL2, or PL3 pore loop types in protomers 2, 3 and 4. Protomers 2-4 are selected for the analysis as both NBDs are in an active (ATP-bound) state in the pre-hydrolysis ClpB structure (PDB-6OAX (8)). Although protomer 5 is also bound to ATP in both NBD1 and NBD2, it was not included in our analysis, since its greater flexibility, due to the weak lateral interface with protomer 6, is overemphasized in the truncated ClpB variant given the absence of the middle domains. Normalized eigenvectors (PCs) and their associated eigenvalues were obtained by diagonalization of the covariance matrices of position fluctuations $\langle \triangle \mathbf{r}_i \cdot \triangle \mathbf{r}_j \rangle$ using Python packages MDTraj (9) and NumPy (10). We compared the similarity of PC motions within the essential subspaces of the wild-type and BB variants by calculating the overlap of two eigenvectors, the cumulative overlap (CO) of one eigenvector of one subspace and eigenvectors of the other subspace and root-mean-square inner product (RMSIP) between eigenvectors of the two subspaces. The overlap between the directions of a wild-type PC and a BB PC is (11)

$$O_{ij} = |\, \mu_i^{wt} \cdot \upsilon_j^{BB} \,|$$

where μ_i^{wt} is the *i^th^* PC of the PL in wild-type ClpB, and ν_j^{BB} is the jth PC of the same PL in the BB variant. The cumulative overlap between the first *k* PCs of the PL in wild-type ClpB and the jth PC of the PL in the BB variant is defined as (12)

$$CO_j(k) = \left(\sum_{i=1}^{k} O_{ij}^2 \right)^{\frac{1}{2}}$$

The CO provides information about the extent to which a single BB PC is represented within the motions captured by the first *k* wild-type PCs. RMSIP measures the overlap between subspaces spanned by the first *m* PCs of wild-type and BB variants by using (12,13)

$$RMSIP(m) = \left(\frac{1}{m} \sum_{j=1}^{m} \sum_{i=1}^{k} O_{ij}^2 \right)^{1/2} = \left[\frac{1}{m} \sum_{j=1}^{m} (CO_j(k)^2) \right]^{\frac{1}{2}}$$

Optimal and suboptimal path analysis

We performed the path analysis in protomers 2-4 (PDB-6OAX (8)) and determined the optimal and suboptimal intra-domain paths traversing from each NBD Walker B region to each pore loop using the *cnapath* function and the Girvan-Newman algorithm (14) implemented in the Bio3D package (15,16). In the allosteric network, each Cα atom represents a node, and network edges are weighted by $w_{ij} = -\log(|C_{ij}|)$. To remove weakly correlated and physically distant residue pairs, we set $|C_{ij}| \geqslant 0.3$ and the Cα -Cα distance $d_{ij} \leqslant 10$ Å (16). The optimal path corresponds to the shortest distance between the "source" (Walker B) and "sink" (pore loop) residue pairs, whereas suboptimal paths are slightly longer paths, excluding the optimal one.

Similarity between probability density distributions is evaluated using the overlapping coefficient (17,18) is $OC = \int \min[p_1(x), p_2(x)]\, dx$, where $p_i(x), i = 1, 2,$ are the probability densities to compare.

Supplemental Figures:

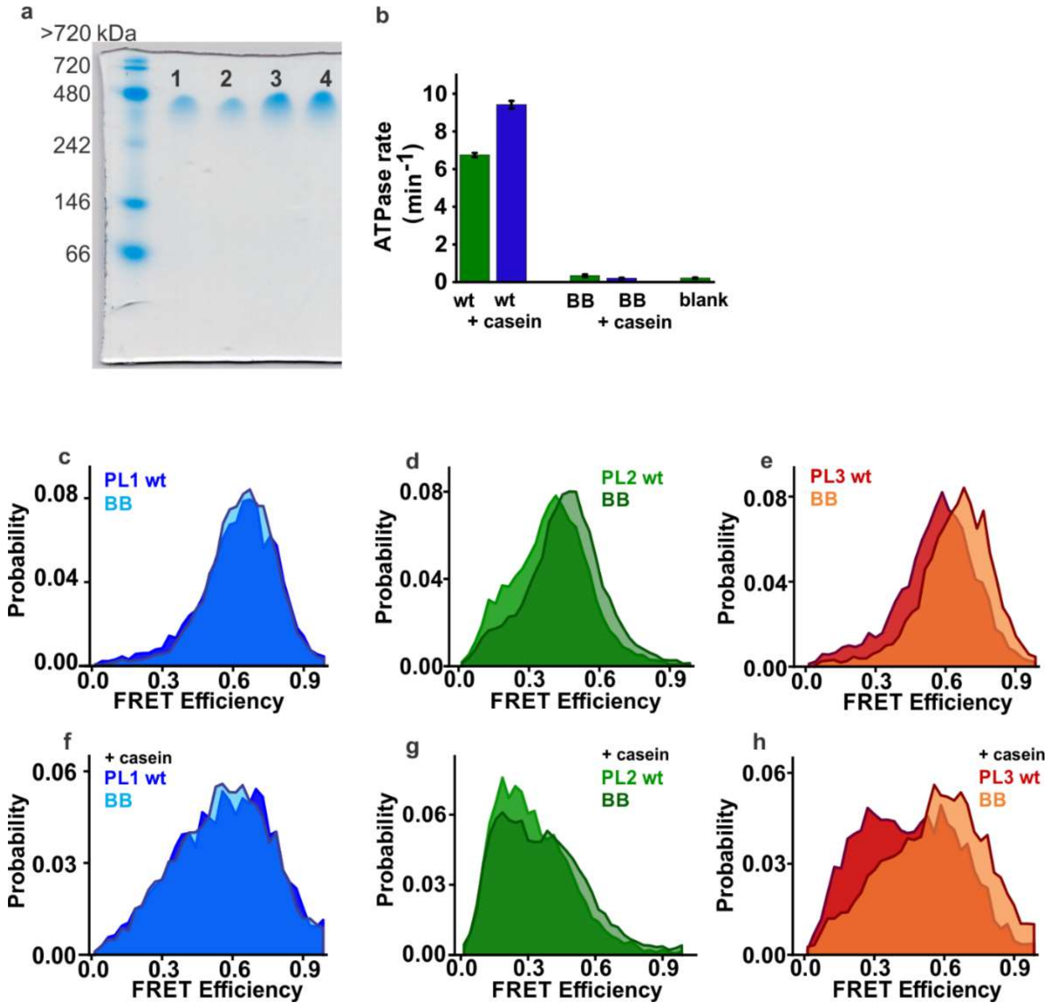

Figure S1. Double-Walker B (BB) mutants of ClpB show correct assembly but abolished ATP hydrolysis rate and display differential behavior in smFRET experiments. (a) Native gel (6% acrylamide), stained with Coomassie Blue. Lanes 1, 2 - unmodified dNClpB (referred to as "ClpB" or "wt") and lanes 3, 4 - its double mutant E271A/E668A (numbering as in full-length *TT* ClpB, and denoted as ‘BB’ here and elsewhere below). Both unmodified wt and BB migrate as a single band in the presence of ATP (run at 4°C, 30 V, 2 mM ATP, 4 mM Mg^{2+}) at the molecular weight ~480 kDa (NativeMark^{TM} Unstained Protein Standard, Thermo Fisher Scientific Inc). This is consistent with their homogeneous assembly into dNClpB hexamers (theoretical MW=484.8 kDa). **(b)** Basal and κ-casein (25 μM) stimulated ATPase activities at 25°C (in green and blue, respectively) of wt dNClpB and BB mutant (standard error, n=5). Blank is recorded without added protein. As expected, the BB mutations completely abolish ATP hydrolysis and casein-induced stimulation. **(c)** FRET efficiency histograms of PL1 and double-Walker B construct. Unmodified PL1 ("wt") is in solid blue and PL1 BB ("BB") in semi-transparent cyan. **(d)** PL2 wt is in solid green and PL2 BB in semi-transparent dark-green. **(e)** PL3 wt is in solid red and PL3 BB in semi-transparent orange. **(f)** PL1 wt and PL1 BB with 25 μM κ-casein. Colors as in (a). **(g)** PL2 wt and PL2 BB with 25 μM κ-casein. Colors as in (b). **(h)** PL3 wt and PL3 BB with 25 μM κ-casein. Colors as in (c). Here and elsewhere below, single-molecule measurements were conducted using 1:100 labeled:unlabeled ClpB with 2 mM ATP.

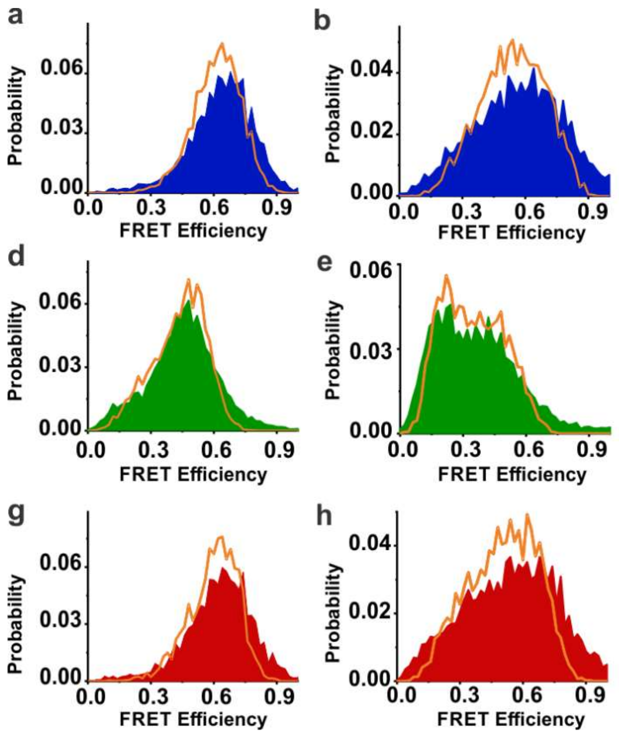

Figure S2. H^{2}MM analysis of Walker BB mutants. Recoloring and segmentation analyses of single-molecule FRET data were carried as previously described (1,3,19). See more details under "Supplemental smFRET Data Analysis Details". **(a,b,d,e,g,h)** Recoloring of FRET-efficiency histograms of PL BB data. Recolored histograms are shown as orange curves. **(a)** PL1 BB. **(b)** PL1 BB + κ-casein (25 μM). **(d)** PL2 BB. **(e)** PL2 BB + κ-casein (25 μM). **(g)** PL3 BB. **(h)** PL3 BB + κ-casein (25 μM). In all cases, the quality of recoloring is comparable to that for PLs wt datasets (see our preceding study (1)), even though a fixed-state model is used instead of free model, with the same FRET efficiency values used for different mutants of the same pore-loop type (as detailed in the section "Supplemental smFRET Data Analysis Details" and in Table S1). Histograms are normalized to the sum of events (>7,000 per measurement) and presented with 50 bins. **(c)** Segmentation analysis of FRET efficiency histogram of PL1 BB + κ-casein (25 μM), **(f)** of PL2 with κ-casein (25 μM), **(i)** of PL3 with κ-casein (25 μM). Segmentation analyses clearly show two states, at low FRET efficiency (green curve) and at high FRET efficiency (dark-red curve).

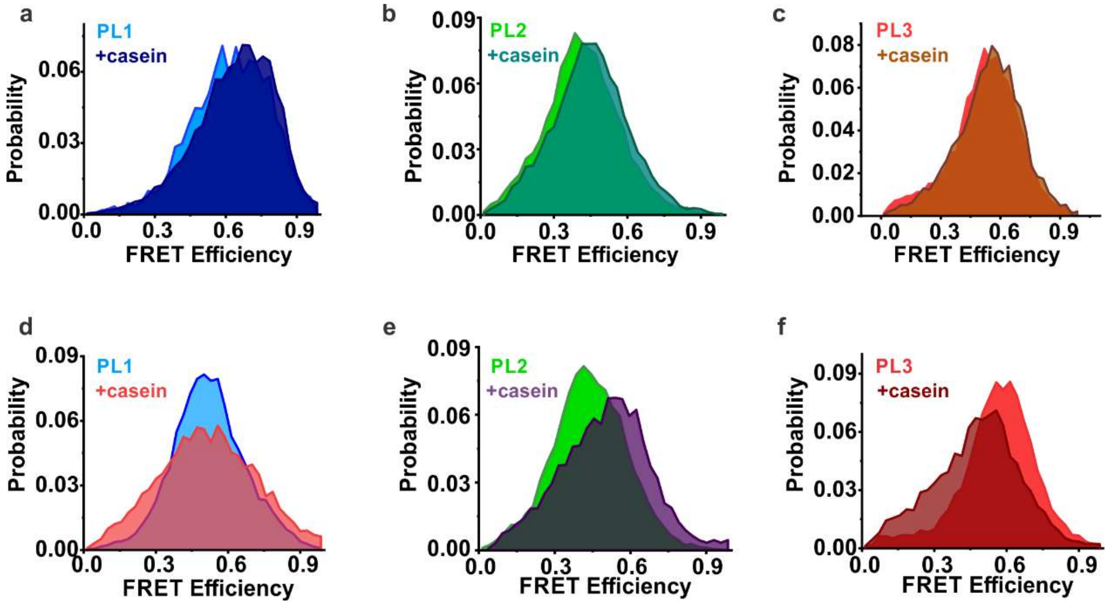

Figure S3. FRET efficiency histograms from experiments under assembly-disfavoring conditions. (a-c) Measurements in the presence of 300 mM KCl, without ATP (apo), (25 mM HEPES, 300 mM KCl, 10 mM $MgCl_2$, 0 mM ATP), where disassembly is expected. Addition of κ-casein (25 μM) causes no shift to low FRET efficiency under these conditions. **(d-f)** Measurements in the absence of added ATP and Mg^{2+} (25 mM HEPES, 25 mM KCl, 0 mM $MgCl_2$, 0 mM ATP), where disassembly is also expected. Addition of κ-casein (25 μM) causes a smaller shift to low FRET efficiency in comparison to the measurements in the presence of 2 mM ATP and 10 mM Mg^{2+}. Casein-induced shift in FRET efficiency histograms is decreased in all three types of the pore loops, especially under high-salt conditions where hexamers of ClpB are expected to be disassembled. These results suggest that casein-induced shift to low FRET efficiency values occurs only in ATP-bound and assembled ClpB.

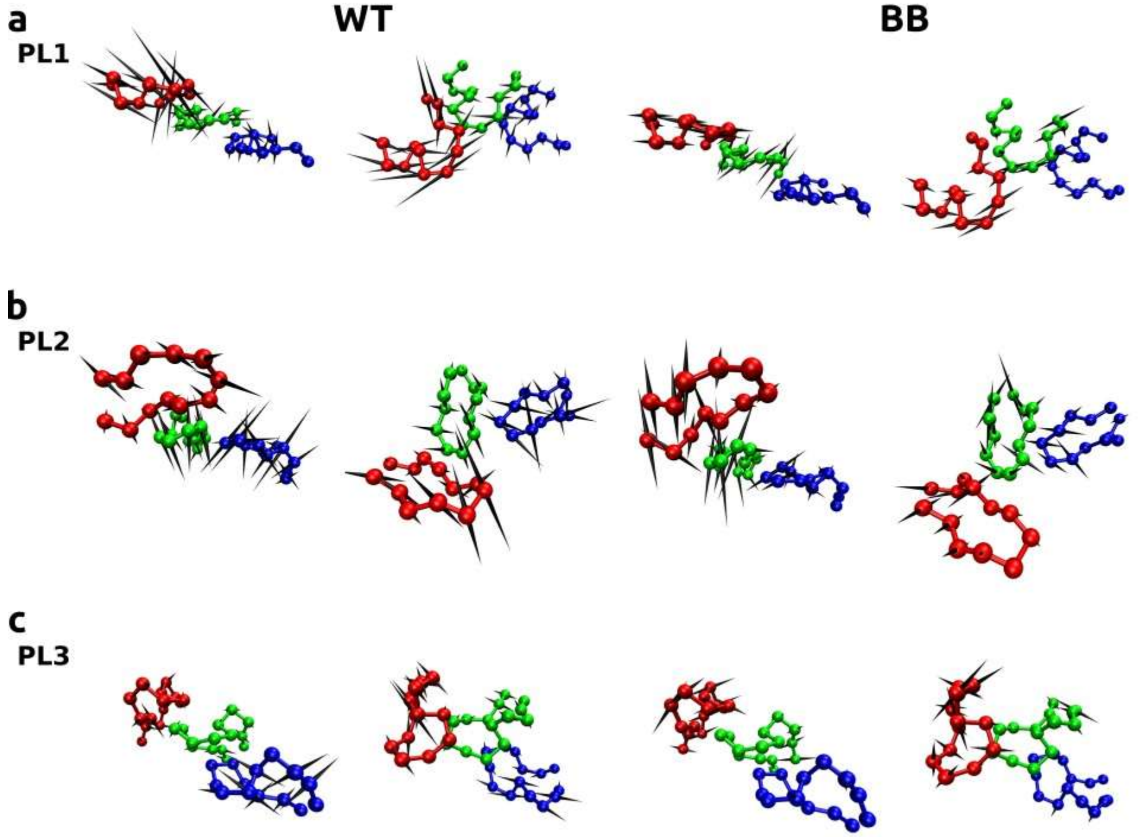

Figure S4. Pore loop motions associated with PC2 eigenvectors. Directions of motion of amino acids of (a) PL1 (b) PL2 (c) PL3 in protomers 2-4 (blue, green and red, respectively) are indicated using spikes for the wild-type (left panels) and BB variants (right panels). Top and side views are shown for both the wild-type and BB variants. The amplitudes of motions, indicated by spike lengths, have been scaled by a factor of 2 for clarity.

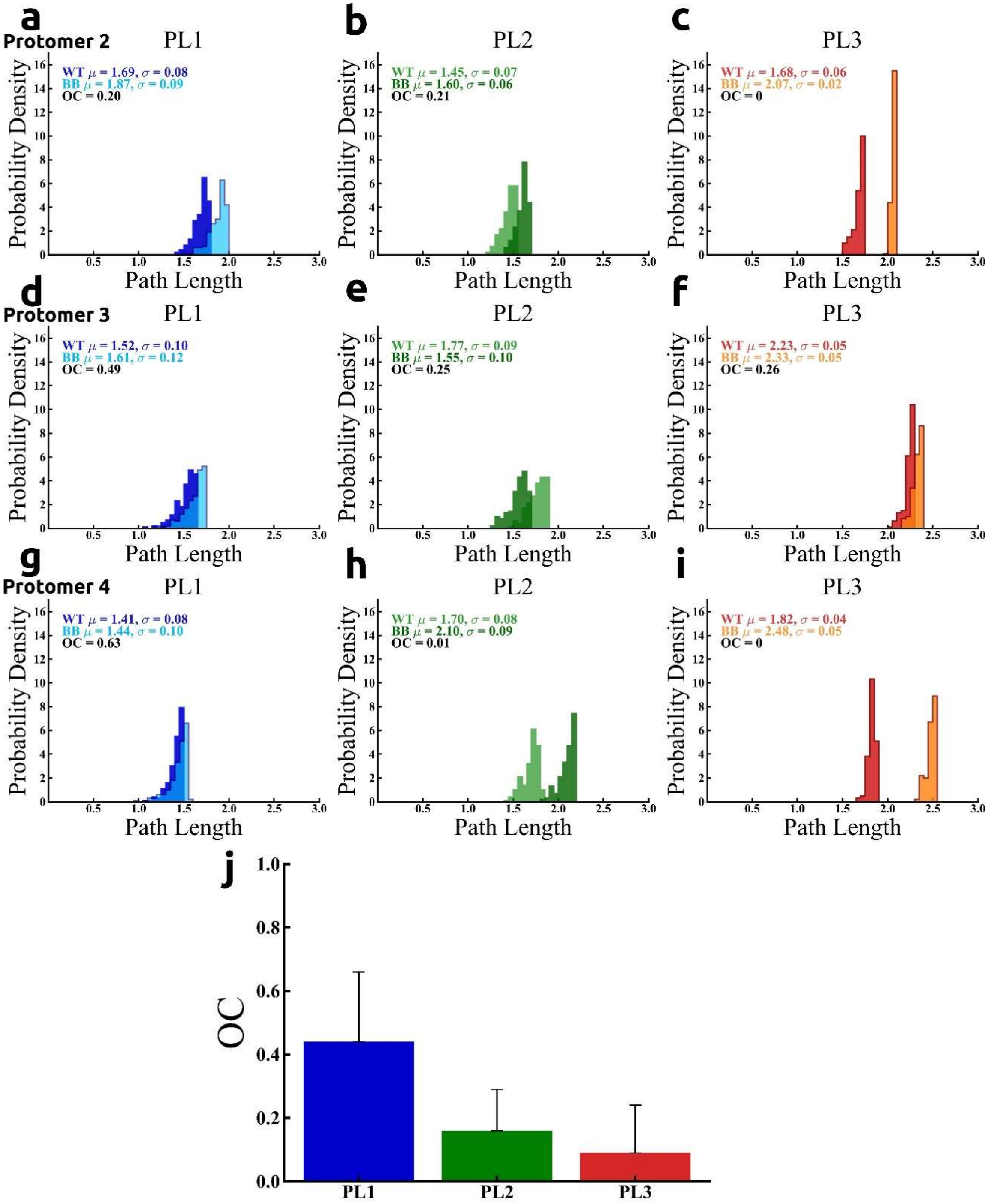

Figure S5. Path length distributions in allosteric signaling in ClpB configurations. Probability density distributions of the 200 shortest path lengths are shown for PL1, PL2, and PL3 wt and BB in protomers **(a-c)** 2, **(d-f)** 3, and **(g-i)** 4. **(j)-(k)** Similarity of path length distributions of PL1-3 are quantified using the average and standard deviation (shown as half error bar), over protomers 2-4,

of **(j)** the overlap coefficient (OC). The effect of perturbation on allosteric communication is weak in PL1, but strong in PL2 and PL3.

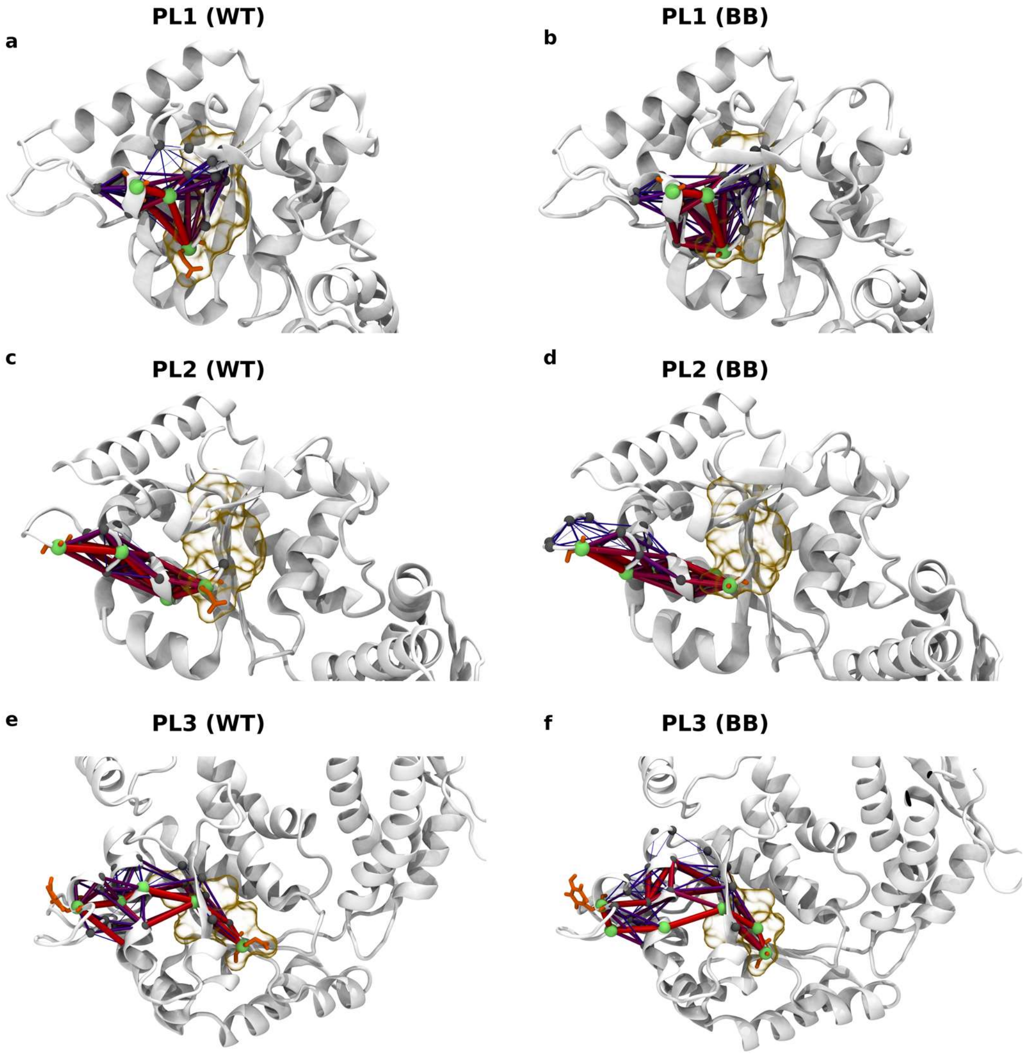

Figure S6. Optimal and suboptimal paths connecting the Walker B regions and pore loops within a ClpB protomer. Paths are shown for **(a-b)** PL1 wt and BB; **(c-d)** PL2 wt and BB; **(e-f)** PL3 wt and BB. The pore loop residues labeled in smFRET experiments, A244 in PL1, A289 in PL2, and Y656 in PL3 (numbering for *E. coli* ClpB) are shown in orange using licorice representation. Optimal paths are slightly perturbed by BB mutations. The ensemble of suboptimal paths (purple), which have longer path lengths, is weakly perturbed by BB mutations for PL1 and strongly perturbed for PL2 and PL3. Line thickness is proportional to the strength of the coupling.

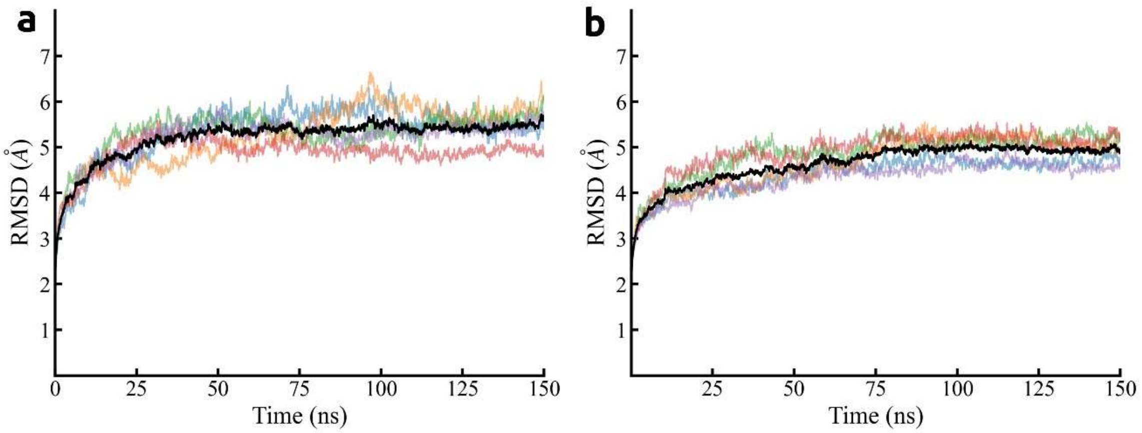

Figure S7. Equilibration of MD trajectories. Root-mean-square deviation calculated as the average over five trajectories (black) and for individual trajectories (color-coded) of **(a)** wild-type ClpB and **(b)** BB variant.

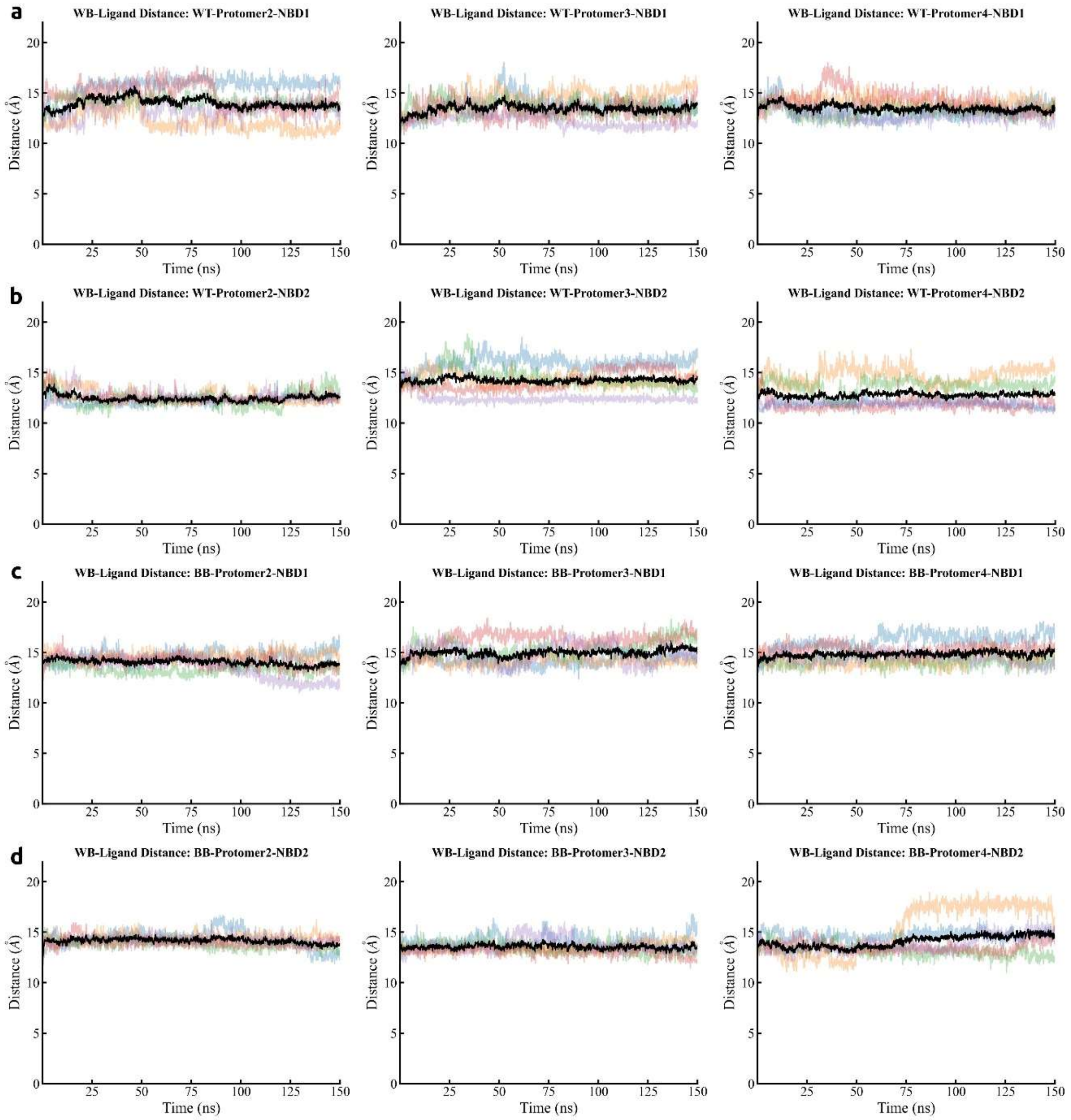

Figure S8. Nucleotide stability within the binding site. The distance between the center of mass of nucleotides and the C_α atom of the Walker B mutation location within the same nucleotide binding site in (a) NBD1 and (b) NBD2 of wild-type ClpB and (c) NBD1 and (d) NBD2 of the BB variant. Time series in individual trajectories are indicated using color-coding and averages are indicated in black.

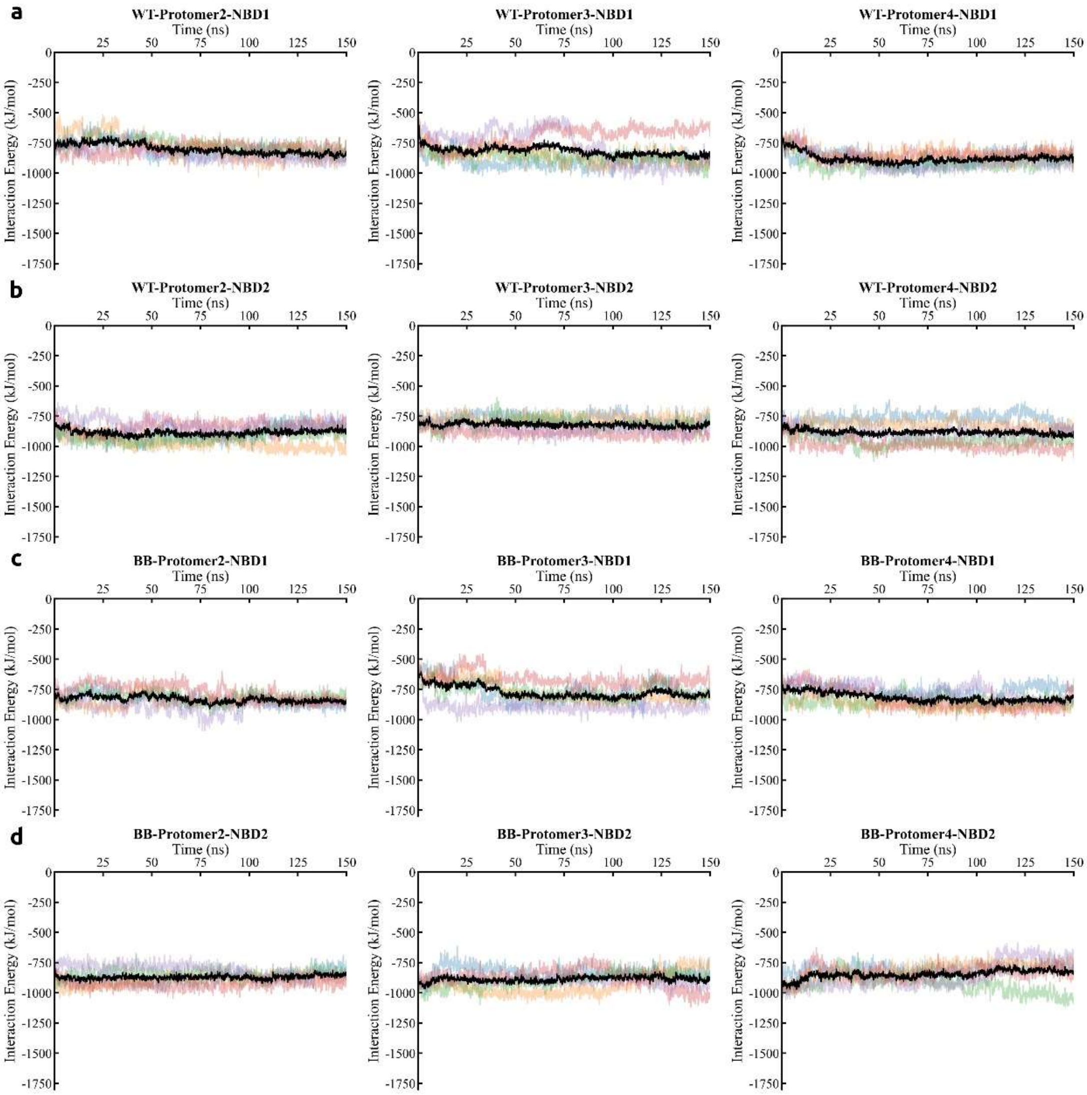

Figure S9. Interaction between nucleotide and ClpB. Interaction energy between (a)-(b) wild-type ClpB and nucleotides in (a) NBD1 and (b) NBD2. (c)-(d) BB variant and nucleotides in (c) NBD1 and (d) NBD2. Time series in individual trajectories are indicated using color-coding and averages are indicated in black.

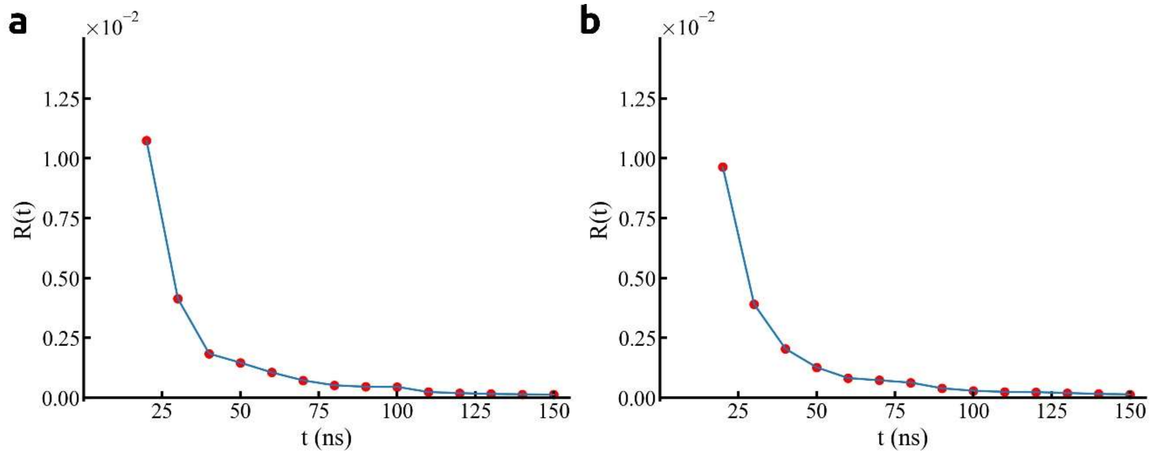

Figure S10. DCCM convergence. Mean-square distance computed over multiple trajectories of **(a)** wild-type **(b)** BB variants.

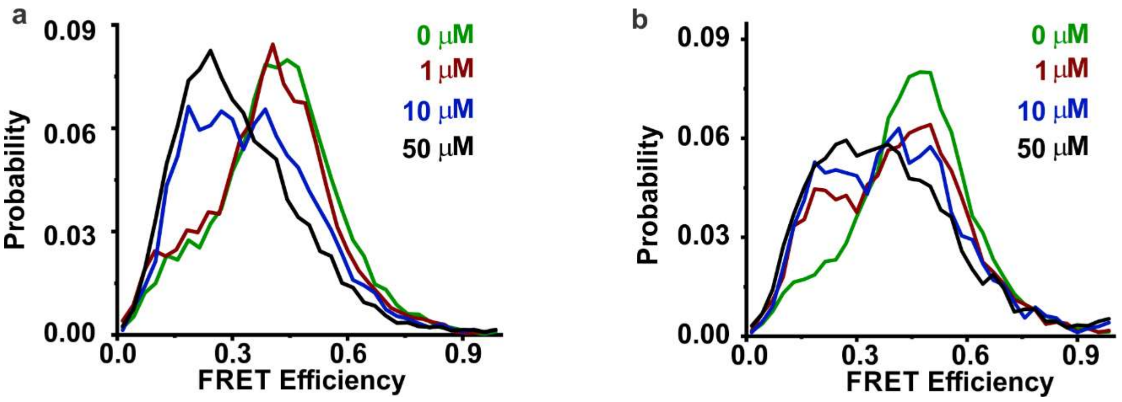

Figure S11. Representative FRET efficiency histograms from single-molecule κ-casein titration assay. (a) PL2 wt. **(b)** PL2 BB. Low-FRET state becomes more populated with increasing concentration of κ-casein. This effect is more prominent for the BB mutant, where a strong change is seen already with 1 μM κ-casein.

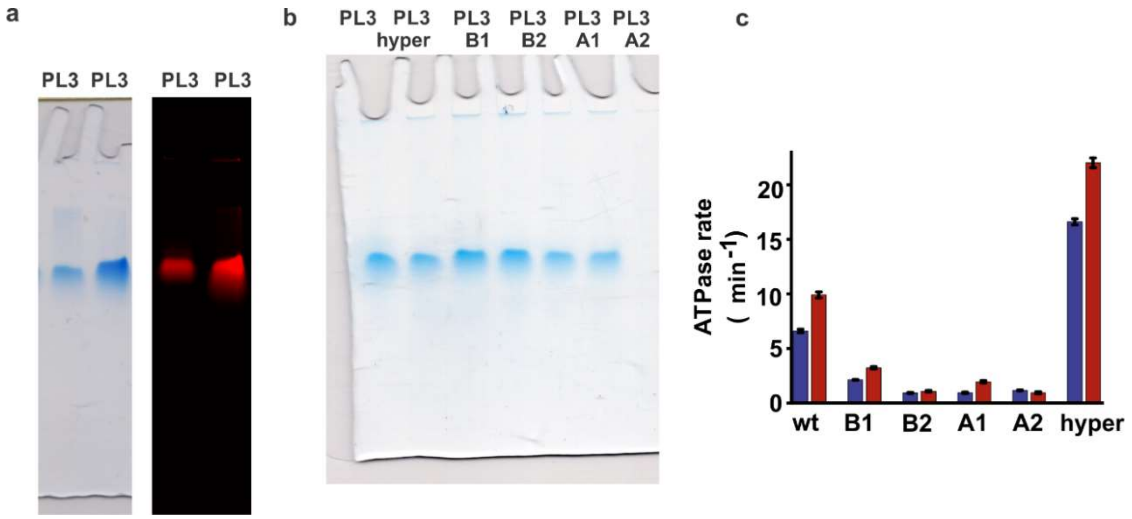

Figure S12. Single mutants of ClpB display correct assembly but modified ATPase activity. **(a)** and **(b)** Native PAGE analysis of 1:100 labeled:unlabeled ClpB samples studied in this work. These typically display single bands, similarly to the results for wt dNClpB (see Fig. S1), indicating homogeneously assembled complexes. **(a)** Representative native gel (6% acrylamide) of 1:100 mixed samples of S359C-Y646C (PL3) with wt. Left: stained with Coomassie Blue. Right: same gel as on the left, imaged with a Typhoon scanner at 532 nm confirms incorporation of fluorescently-labelled molecules into the sample following the mixing procedure. Run in the presence of ATP (at 4°C, 30 V, 2 mM ATP, 4 mM Mg^{2+}). **(b)** Representative native gel (6% acrylamide, run and stained as in **(a)**) of 1:100 mixed S359C-Y646C (PL3) sample and its single mutants. The single mutations here and elsewhere below are as follows: K347A (‘hyper’), E271A (‘B1’), E668A (‘B2’), K204T (‘A1’) and K601A (‘A2’), with numbering as in the full-length *TT* ClpB. Note that here and elsewhere, the A/B mutations are numbered according to the NBD that bears the mutation (1 or 2). **(c)** ATPase activity and casein-induced enhancement of unlabeled cysteine-less single mutants of ClpB. Background-corrected basal (blue bars) and κ-casein (25 μM) stimulated (red bars) ATPase activities at 25°C of wt (dNClpB) and mutants (standard error, n=5). Hyperactive mutation shows 2.5x higher basal rate than wt, and single Walker A and B are characterized by decreased basal ATPase activity. Most of the mutants show κ-casein-induced activity enhancement.

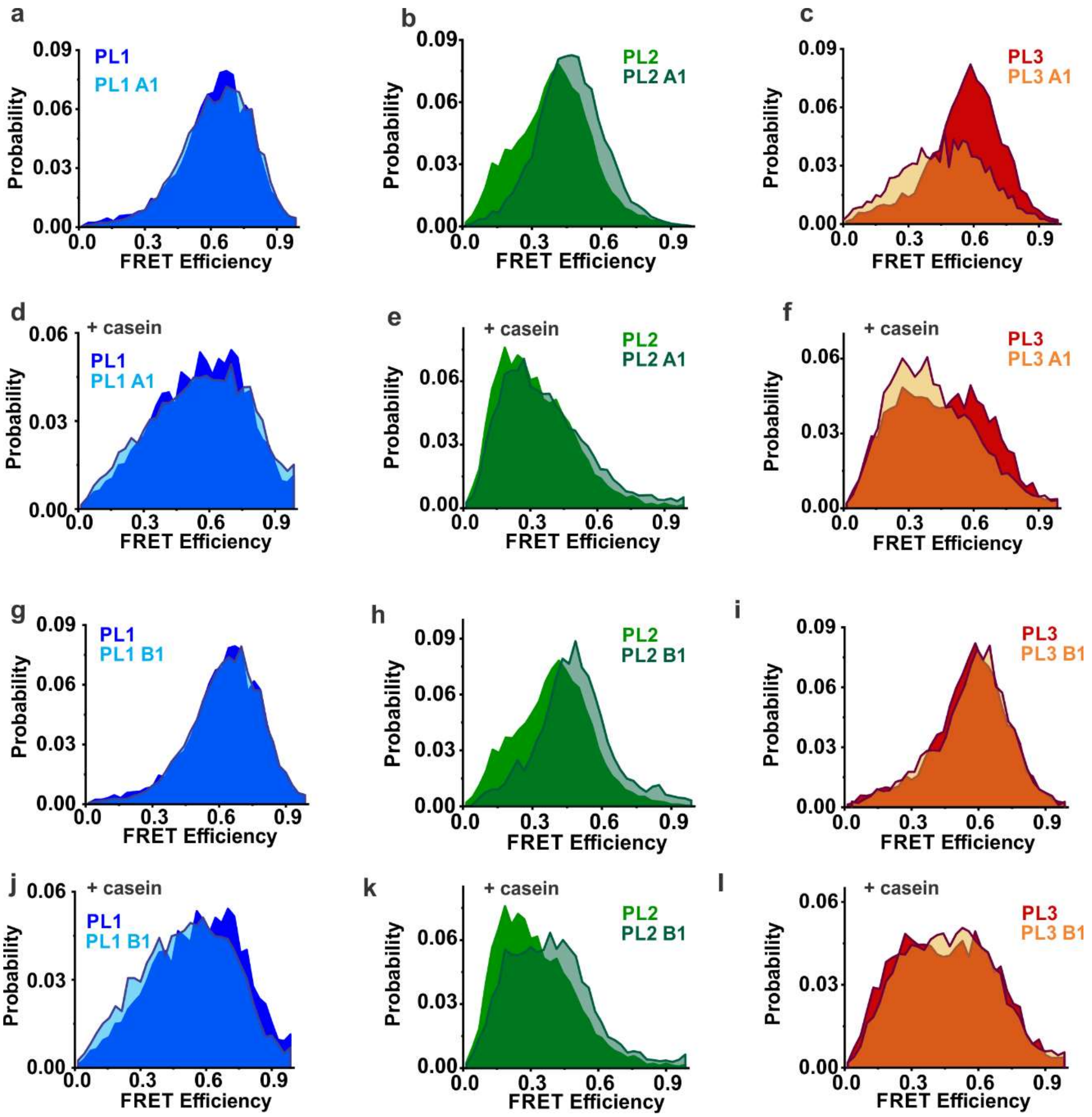

Figure S13. FRET efficiency histograms of NBD1 mutants. PL1, PL2 and PL3 without mutations, and Walker A1 (K204T) and Walker B1 (E271A) results. "+ casein" denotes measurements with κ-casein (25 μM). Color scheme as in Fig. S1.

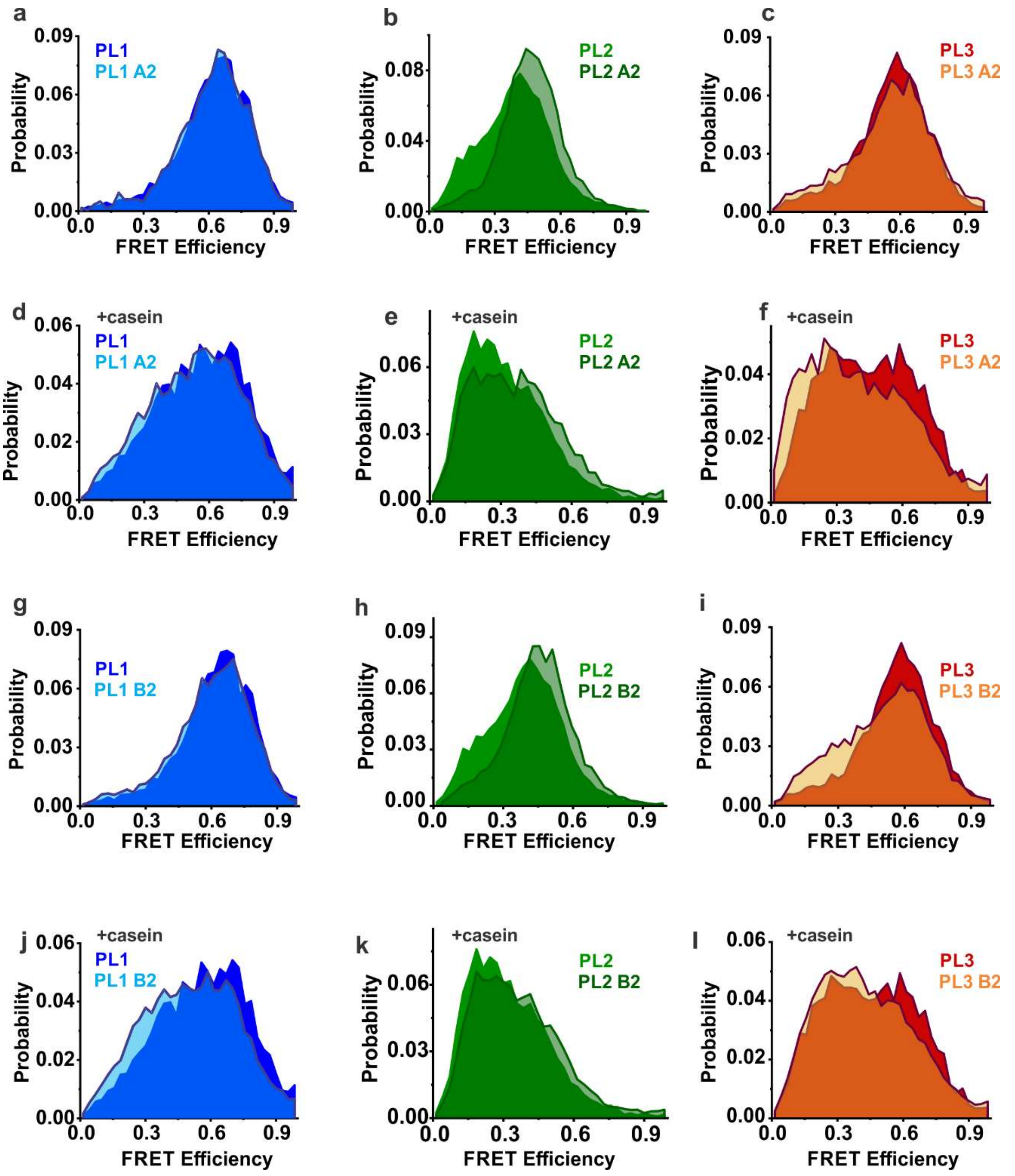

Figure S14. FRET efficiency histograms of NBD2 mutants. PL1, PL2 and PL3 without mutations, and Walker A2 (K601T) and Walker B2 (E668A) results. "+ casein" denotes measurements with κ-casein (25 μM). Color scheme as in Fig. S1.

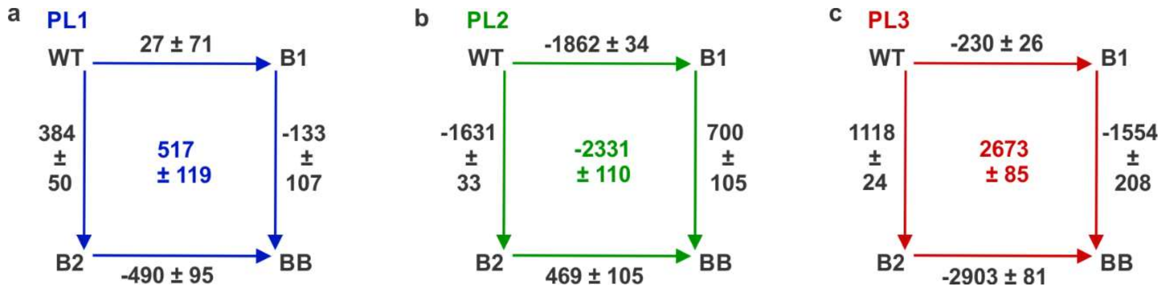

Figure S15. Double-mutant cycles (20) for PL1, PL2 and PL3 without κ-casein. The values along the sides are the free energy changes associated with the indicated mutations, $\Delta\Delta G_i$ (in J.mol^{-1}), calculated from the H^{2}MM-derived K_is as detailed in Materials and Methods, main text. In these cycles, the $\Delta\Delta G_i$ values along the opposite edges are unequal: $\Delta\Delta G_{i_{WT\rightarrow B1}} \neq \Delta\Delta G_{i_{B2\rightarrow BB}}$ and $\Delta\Delta G_{i_{WT\rightarrow B2}} \neq \Delta\Delta G_{i_{B1\rightarrow BB}}$. This indicates thermodynamic coupling of the effect of the two mutations on pore-loop conformations. The coupling energies for each pore-loop type (Materials and Methods, main text) are shown at the centres of the cycles. Error is from the propagation of the standard error in K_i.

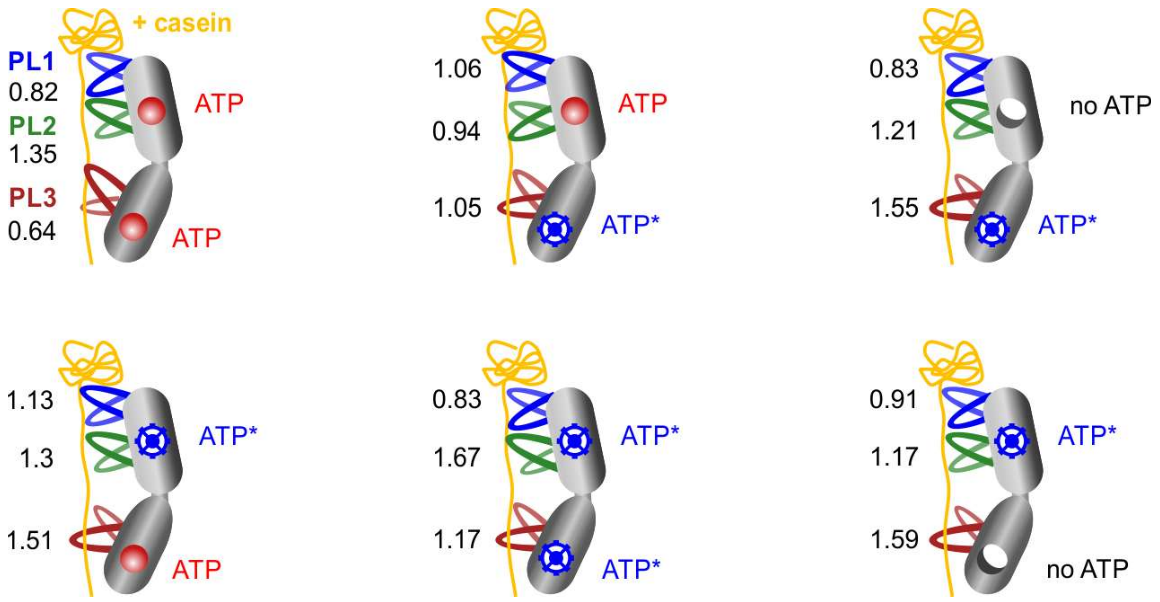

Figure S16. Summary of all smFRET results suggests an ATP-dependent modulation of pore-loop dynamics. As in Figure 7 (main text), states of ClpB pore loops are schematically shown, with bound κ-casein in yellow, and PL1, PL2 and PL3 in blue, green and dark-red, respectively. The ATPase states of the NBDs are depicted as follows: red circles are bound ATP molecules not undergoing hydrolysis (ATP arrested state), blue wheels – bound ATP undergoing hydrolysis, which corresponds to a mixture of ATP/ADP, and empty circles – unbound or apo state. To note, these apo states are likely to be very transient under our experimental conditions (excess ATP at 2 mM). Nevertheless, they represent important states within the ATPase cycle (essential precursors for the ATP re-binding to occur), and their presence in ClpB was previously experimentally captured by cryo-EM (21). As in Figure 7 (main text), PLs can favor either an up or a down conformation, or visit both states with almost equal probability, depending on the ATPase state of the NBDs. The size of the PLs in the scheme reflects their state occupancy based on the H^2MM analysis, and the numbers are H^2MM-derived equilibrium coefficients, K_is.

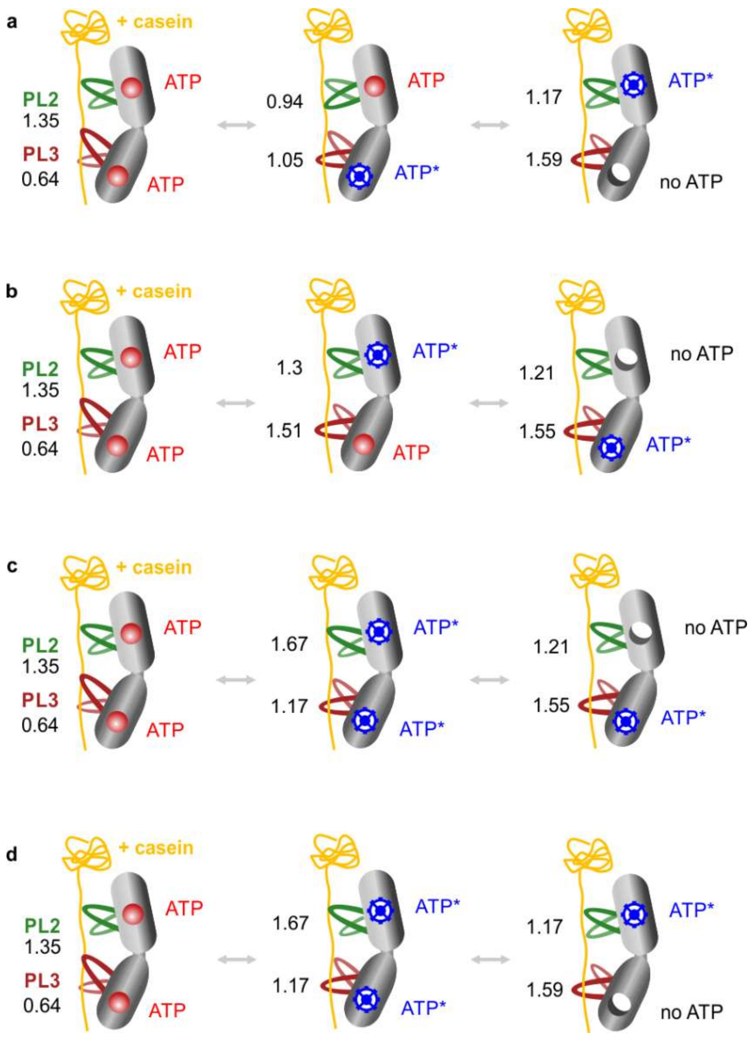

Figure S17. Examples of potential routes leading to substrate-protein translocation by ClpB. (a-d) These routes can be considered under a few assumptions: 1) The order of events is based on the ATP hydrolysis reaction (ATP binds → ATP hydrolysis event → nucleotide release results in apo state → ATP re-binds). 2) There is no preference for the order of ATP binding/hydrolysis happening in NBD1 or in NBD2. 3) A translocation event occurs upon a change in the equilibrium position of PL3 from up to down state, happening within a single protomer of ClpB. During this transition, PL3 is either pulling on the substrate-protein, or is acting as a pawl to guide its movement, as discussed in the main text. Here, there is no requirement for any specific conformations of PL2 relative to PL3. To point out, the functioning protomer still needs to be embedded into a hexamer to ensure correct ATP binding and hydrolysis. There is no information on the states of neighboring protomers because only intra-protomer dynamics are being measured in our assays, and therefore substrate-protein translocation driven by the movements of two or more protomers within a hexamer cannot be inferred. For simplicity, PL1 is omitted since this pore-loop type did not show strong ATP-dependent modulations. The size of the PLs on the scheme reflects their state occupancy from the H^2MM analysis, and the numbers are H^2MM-derived equilibrium coefficients, K_is.

Supplemental Movies:

Movie SM1. Motions associated with the principal component PC1 of PL1 loops in wild-type ClpB (left) and in the BB variant (right).

Movie SM2. Motions associated with the principal component PC1 of PL2 loops in wild-type ClpB (left) and in the BB variant (right).

Movie SM3. Motions associated with the principal component PC1 of PL3 loops in wild-type ClpB (left) and in the BB variant (right).

Supplemental Data Summary Tables:

Table S1. FRET efficiency values of the two pore loop states as obtained from the global H^2MM analysis of wild-type pore-loop constructs, measured with or without κ-casein (1)**.**

**Pore loop**	**FRET value** State 1	**FRET value** State 2
**PL1**	0.240	0.767
**PL2**	0.182	0.536
**PL3**	0.212	0.699

Table S2. Cumulative overlap of the top 2 principal components of pore loops in the BB variant over the top 10 principal components of the corresponding pore loops in wild-type ClpB.

Cumulative Overlap	BB-PL1-PC1	BB-PL1-PC2	BB-PL2-PC1	BB-PL2-PC2	BB-PL3-PC1	BB-PL3-PC2
wt-PC1	0.8451	0.0782	0.1004	0.0926	0.1158	0.0790
wt-PC2	0.8452	0.5416	0.2260	0.5088	0.2556	0.0865
wt-PC3	0.8777	0.6099	0.2281	0.5589	0.2677	0.1110
wt-PC4	0.8778	0.6113	0.3076	0.5792	0.5208	0.1522
wt-PC5	0.8913	0.6275	0.3653	0.5817	0.5208	0.3335
wt-PC6	0.8915	0.7394	0.4602	0.7048	0.5225	0.3859
wt-PC7	0.9641	0.7397	0.6203	0.7070	0.6305	0.4611
wt-PC8	0.9694	0.7560	0.6642	0.7070	0.6766	0.5630
wt-PC9	0.9700	0.7801	0.8026	0.7421	0.6922	0.6519
wt-PC10	0.9734	0.7824	0.8172	0.7678	0.7410	0.6881

Table S3: Optimal paths between Walker B sites and pore loops in NBD1, PL1 and PL2, in wild type ClpB and BB mutants, derived from the MD simulations of *E. coli* ClpB.

Protomer	PL1	
	Wild-type	BB
2	A279 → M283 → M242 → G243 → A244	A279 → M242 → G243 → A244
3	A279 → A241 → A244	A279 → M242 → G243 → A244
4	A279 → A241 → A244	A279 → A241 → A244

Protomer	PL2	
	Wild-type	BB
2	A279 → M283 → A286 → K288 → A289	A279 → L280 → V284 → G285 → K288 → A289
3	A279 → M283 → A287 → K288 → A289	A279 → L280 → V284 → K288 → A289
4	A279 → L280 → H281 → A286 → A289	A279 → L280 → V284 → G285 → A289

Table S4: Optimal paths between Walker B sites and pore loops in NBD2, PL3, in wild type ClpB and BB mutants, derived from the MD simulations of *E. coli* ClpB.

Protomer	PL3	
	Wild-type	BB
2	A678 → D677 → I632 → L646 → V647 → G648 → Y656	A678 → M716 → I715 → V714 → V713 → T712 → F709 → V707 → D701 → Y656
3	A678 → D677 → I632 → L646 → A649 → Y656	A678 → D677 → I632 → L646 → A647 → Y656
4	A678 → I632 → Y661 → G660 → Y656	A678 → D677 → I632 → R645 → A649 → Y656

Table S5. Substrate κ-casein binding constants to individual pore-loop mutants, derived from H^2MM analysis of single-molecule κ-casein titration measurements.

Sample	K_d (µM)	Error (from fit)*	F_{max}	Error (from fit)
**PL1 wt**	2.9	0.5	0.56	0.02
**PL1 BB**	2.0	0.4	0.57	0.04
**PL2 wt**	11.3	3.0	0.83	0.10
**PL2 BB**	0.3	0.1	1.03	0.03
**PL3 wt**	0.9	0.4	0.74	0.06
**PL3 BB**	1.6	0.7	0.97	0.09

*Data, presented in Fig. 5 (main text) were fitted to a simple binding isotherm $y = 1 + \frac{\text{Fmax} \times [\text{casein}]}{(K_d + [casein])}$

where [casein] is κ-casein concentration (µM) and F_{max} is the maximum P1(+ casein)/P1(- casein).

Tables S6: H^{2}MM Analysis Outputs. Average state populations, equilibrium constants, K_i and state-to-state transition rates, k^i, (in s^{-1}) for each pore loop type (i), compared to dwell time analysis.

PL1: WT and double mutants.

Pore loop sampe+2 mM ATP	State Populations		K_i	k^i_{12}		k^i_{21}	
	State 1	State 2		H^{2}MM	Dwell time	H^{2}MM	Dwell time
PL1	0.32 ± 0.01	0.68 ± 0.01	0.46 ± 0.02	44370 ± 1750	42360 ± 1700	20640 ± 1780	23630 ± 470
PL1 + κ-casein	0.45 ± 0.01	0.55 ± 0.01	0.83 ± 0.02	17670 ± 2230	21490 ± 1190	14730 ± 1720	19160 ± 1070
PL1 BB	0.31 ± 0.02	0.69 ± 0.02	0.45 ± 0.05	47580 ± 5990	42700 ± 4130	22460 ± 1900	23160 ± 770
PL1 BB + κ-casein	0.45 ± 0.01	0.55 ± 0.01	0.82 ± 0.02	21350 ± 810	22900 ± 470	17470 ± 560	20460 ± 250

The errors (standard deviation) were calculated from at least three measurements. Here and elsewhere below, "+ κ-casein" is with 25 µM κ-casein.

PL2: WT and double mutants.

Pore loop sampe+2 mM ATP	State Populations		K_i	k^i_{12}		k^i_{21}	
	State 1	State 2		H^{2}MM	Dwell time	H^{2}MM	Dwell time
PL2	0.44 ± 0.01	0.56 ± 0.01	0.79 ± 0.02	11230 ± 880	13090 ± 90	8890 ± 510	11100 ± 290
PL2 + κ-casein	0.62 ± 0.01	0.38 ± 0.01	1.67 ± 0.04	5950 ± 280	9500 ± 310	9910 ± 370	12180 ± 200
PL2 BB	0.33 ± 0.02	0.67 ± 0.02	0.49 ± 0.05	10700 ± 1380	13540 ± 180	5260 ± 150	9000 ± 80
PL2 BB + κ-casein	0.57 ± 0.02	0.43 ± 0.02	1.35 ± 0.1	4680 ± 190	6210 ± 520	6230 ± 200	7440 ± 40

The errors (standard deviation) were calculated from at least two measurements.

PL3: WT and double mutants.

Pore loop sampe+2 mM ATP	State Populations		K_i	k^i_{12}		k^i_{21}	
	State 1	State 2		H^{2}MM	Dwell time	H^{2}MM	Dwell time
PL3	0.34 ± 0.01	0.66 ± 0.01	0.50 ± 0.02	28300 ± 4100	29730 ± 3780	14250 ± 2020	18430 ± 1760
PL3 + κ-casein	0.54 ± 0.01	0.46 ± 0.01	1.17 ± 0.06	9650 ± 240	13470 ± 560	11330 ± 250	13680 ± 80
PL3 BB	0.20 ± 0.01	0.80 ± 0.01	0.24 ± 0.02	33890 ± 3700	26540 ± 1170	8280 ± 1290	11330 ± 980
PL3 BB + κ-casein	0.39 ± 0.01	0.61 ± 0.01	0.64 ± 0.04	13440 ± 580	15090 ± 240	8530 ± 110	12080 ± 710

The errors (standard deviation) were calculated from at least two measurements.

<u>PL1:</u> Single-NBD mutants.

Pore loop sampe+2 mM ATP	State Populations		K_i	k^i_{12}		k^i_{21}	
	State 1	State 2		H²MM	Dwell time	H²MM	Dwell time
**PL1 A1**	0.31 ± 0.01	0.69 ± 0.01	0.45 ± 0.03	46490 ± 1330	41280 ± 340	20760 ± 1960	23480 ± 1000
**PL1 A1 + κ-casein**	0.45 ± 0.01	0.55 ± 0.01	0.83 ± 0.05	15140 ± 920	20560 ± 770	12540 ± 70	18530 ± 80
**PL1 A2**	0.32 ± 0.01	0.68 ± 0.01	0.47 ± 0.02	41750 ± 290	42090 ± 400	19550 ± 600	24020 ± 420
**PL1 A2 + κ-casein**	0.48 ± 0.01	0.52 ± 0.01	0.91 ± 0.05	19300 ± 1980	26040 ± 1550	17570 ± 850	24100 ± 230
**PL1 B1**	0.32 ± 0.01	0.68 ± 0.01	0.47 ± 0.03	48460 ± 830	42090 ± 750	22770 ± 1930	24240 ± 1430
**PL1 B1 + κ-casein**	0.51 ± 0.01	0.49 ± 0.01	1.06 ± 0.02	14640 ± 4640	22530 ± 3130	15450 ± 4580	23220 ± 2680
**PL1 B2**	0.35 ± 0.01	0.65 ± 0.01	0.54 ± 0.01	41090 ± 7610	41100 ± 4230	22260 ± 3630	25700 ± 1950
**PL1 B2 + κ-casein**	0.53 ± 0.01	0.47 ± 0.01	1.13 ± 0.07	15010 ± 550	22860 ± 800	16900 ± 380	23820 ± 540

The errors (standard deviation) were calculated from at least two measurements.

<u>PL1:</u> Other conditions

Pore loop sampe+2 mM ATP	State Populations		K_i	k^i_{12}		k^i_{21}	
	State 1	State 2		H²MM	Dwell time	H²MM	Dwell time
**PL1 hyper (K347A)**	0.28 ± 0.01	0.72 ± 0.01	0.39 ± 0.01	46920 ± 90	43540 ± 1930	18320 ± 730	22060 ± 1170
**PL1 hyper (K347A) + κ-casein**	0.41 ± 0.01	0.59 ± 0.01	0.69 ± 0.03	19050 ± 3330	24120 ± 4780	13050 ± 1650	19330 ± 2760
**PL1 apo 300 mM KCl**	0.35 ± 0.03	0.65 ± 0.03	0.53 ± 0.08	45330 ± 7670	43630 ± 4050	23780 ± 410	27140 ± 330
**PL1 apo 300 mM KCl + κ-casein**	0.32 ± 0.03	0.68 ± 0.03	0.47 ± 0.06	40140 ± 4320	39120 ± 3990	18710 ± 220	23170 ± 360
**PL1 apo no Mg^{2+}**	0.49 ± 0.02	0.51 ± 0.02	0.98 ± 0.09	49570 ± 110	39400 ± 430	48590 ± 4530	38700 ± 2290
**PL1 apo no Mg^{2+} + κ-casein**	0.52 ± 0.01	0.48 ± 0.01	1.10 ± 0.02	21480 ± 1300	22480 ± 1130	23600 ± 1930	23490 ± 1074

The errors (standard deviation) were calculated from at least two measurements.

PL2: Single-NBD mutants.

Pore loop sampe+2 mM ATP	State Populations		K_i	k^i_{12}		k^i_{21}	
	State 1	State 2		H^2MM	Dwell time	H^2MM	Dwell time
**PL2 A1**	0.26 ± 0.003	0.74 ± 0.003	0.35 ± 0.01	23920 ± 1730	19450 ± 860	8390 ± 480	10620 ± 310
**PL2 A1 + κ-casein**	0.55 ± 0.02	0.45 ± 0.02	1.21 ± 0.11	6410 ± 580	9390 ± 500	7730 ± 30	11080 ± 50
**PL2 A2**	0.25 ± 0.005	0.75 ± 0.005	0.33 ± 0.01	25150 ± 790	18110 ± 370	8360 ± 60	10570 ± 120
**PL2 A2 + κ-casein**	0.54 ± 0.02	0.46 ± 0.02	1.17 ± 0.09	5870 ± 50	9720 ± 30	6850 ± 440	11070 ± 70
**PL2 B1**	0.27 ± 0.01	0.73 ± 0.01	0.37 ± 0.01	16210 ± 2490	16430 ± 310	6000 ± 770	9990 ± 450
**PL2 B1 + κ-casein**	0.48 ± 0.03	0.52 ± 0.03	0.94 ± 0.1	8970 ± 780	11570 ± 560	8340 ± 270	11410 ± 130
**PL2 B2**	0.29 ± 0.01	0.71 ± 0.01	0.41 ± 0.01	18980 ± 800	15130 ± 180	7740 ± 140	10110 ± 10
**PL2 B2 + κ-casein**	0.56 ± 0.02	0.44 ± 0.02	1.3 ± 0.1	6580 ± 790	10710 ± 860	8340 ± 390	13610 ± 2330

The errors (standard deviation) were calculated from at least two measurements.

PL2: Other conditions

Pore loop sampe+2 mM ATP	State Populations		K_i	k^i_{12}		k^i_{21}	
	State 1	State 2		H^2MM	Dwell time	H^2MM	Dwell time
**PL2 hyper (K347A)**	0.22 ± 0.01	0.78 ± 0.01	0.28 ± 0.01	28200 ± 1270	22660 ± 400	8030 ± 80	11050 ± 670
**PL2 hyper (K347A) + κ-casein**	0.46 ± 0.005	0.54 ± 0.005	0.87 ± 0.02	8460 ± 170	10970 ± 150	7350 ± 290	11160 ± 400
**PL2 apo 300 mM KCl**	0.37 ± 0.01	0.63 ± 0.01	0.59 ± 0.02	16480 ± 730	9740 ± 70	18240 ± 800	12210 ± 5
**PL2 apo 300 mM KCl + κ-casein**	0.33 ± 0.01	0.67 ± 0.01	0.50 ± 0.02	17500 ± 620	8720 ± 50	16590 ± 240	10800 ± 140
**PL2 apo no Mg^{2+}**	0.33 ± 0.005	0.67 ± 0.005	0.49 ± 0.01	18300 ± 1200	16900 ± 630	8950 ± 390	10640 ± 290
**PL2 apo no Mg^{2+} + κ-casein**	0.26 ± 0.01	0.74 ± 0.01	0.36 ± 0.01	9280 ± 460	10900 ± 1100	3300 ± 280	7400 ± 440

The errors (standard deviation) were calculated from at least two measurements.

<u>PL3:</u> Single-NBD mutants.

Pore loop sampe+2 mM ATP	State Populations		K_i	k^i_{12}		k^i_{21}	
	State 1	State 2		H^2MM	Dwell time	H^2MM	Dwell time
**PL3 A1**	0.48 ± 0.01	0.52 ± 0.01	0.91 ± 0.04	19780 ± 1940	16840 ± 3	17950 ± 1020	16060 ± 350
**PL3 A1 + κ-casein**	0.61 ± 0.01	0.39 ± 0.01	1.55 ± 0.07	11860 ± 180	11890 ± 210	18370 ± 550	15620 ± 230
**PL3 A2**	0.36 ± 0.01	0.64 ± 0.01	0.57 ± 0.03	19770 ± 420	17400 ± 690	11200 ± 430	12210 ± 500
**PL3 A2 + κ-casein**	0.61 ± 0.02	0.39 ± 0.02	1.59 ± 0.15	5500 ± 180	9200 ± 90	8750 ± 540	11080 ± 140
**PL3 B1**	0.31 ± 0.01	0.69 ± 0.01	0.46 ± 0.01	26780 ± 3700	21850 ± 1310	12240 ± 1310	13030 ± 170
**PL3 B1 + κ-casein**	0.51 ± 0.01	0.49 ± 0.01	1.05 ± 0.05	15030 ± 400	13170 ± 630	15820 ± 370	13730 ± 400
**PL3 B2**	0.44 ± 0.01	0.56 ± 0.01	0.79 ± 0.03	13570 ± 790	14320 ± 370	10780 ± 280	12060 ± 50
**PL3 B2 + κ-casein**	0.60 ± 0.01	0.40 ± 0.01	1.51 ± 0.04	9840 ± 50	11250 ± 60	14860 ± 310	14000 ± 210

The errors (standard deviation) were calculated from at least two measurements.

<u>PL3:</u> Other conditions

Pore loop sampe+2 mM ATP	State Populations		K_i	k^i_{12}		k^i_{21}	
	State 1	State 2		H^2MM	Dwell time	H^2MM	Dwell time
**PL3 hyper (K347A)**	0.25 ± 0.02	0.75 ± 0.02	0.33 ± 0.03	45400 ± 1670	28420 ± 380	14730 ± 900	13590 ± 40
**PL3 hyper (K347A) + κ-casein**	0.41 ± 0.004	0.59 ± 0.004	0.69 ± 0.01	17830 ± 1430	14280 ± 390	12290 ± 780	13350 ± 440
**PL3 apo 300 mM KCl**	0.38 ± 0.03	0.62 ± 0.03	0.60 ± 0.07	35970 ± 5420	35850 ± 2760	21500 ± 660	23800 ± 10
**PL3 apo 300 mM KCl + κ-casein**	0.36 ± 0.01	0.64 ± 0.01	0.56 ± 0.02	37070 ± 840	35770 ± 190	20770 ± 1030	23510 ± 490
**PL3 apo no Mg^{2+}**	0.32 ± 0.02	0.68 ± 0.02	0.48 ± 0.05	41520 ± 1710	27070 ± 380	19910 ± 2830	14910 ± 90
**PL3 apo no Mg^{2+} + κ-casein**	0.49 ± 0.01	0.51 ± 0.01	0.96 ± 0.02	18670 ± 380	16260 ± 2840	17880 ± 770	15440 ± 2330

The errors (standard deviation) were calculated from at least two measurements.

Supplemental References:

1. Mazal, H., M. Iljina, I. Riven, and G. Haran. 2021. Ultrafast pore-loop dynamics in a AAA+ machine point to a Brownian-ratchet mechanism for protein translocation. *Sci Adv*. 7(36):eabg4674, doi: 10.1126/sciadv.abg4674.
2. Pirchi, M., R. Tsukanov, R. Khamis, T. E. Tomov, Y. Berger, D. C. Khara, H. Volkov, G. Haran, and E. Nir. 2016. Photon-by-Photon Hidden Markov Model Analysis for Microsecond Single-Molecule FRET Kinetics. *J. Phys. Chem. B.* 120(51):13065-13075, doi: 10.1021/acs.jpcb.6b10726.
3. Aviram, H. Y., M. Pirchi, H. Mazal, Y. Barak, I. Riven, and G. Haran. 2018. Direct observation of ultrafast large-scale dynamics of an enzyme under turnover conditions. *Proc Natl Acad Sci U S A*. 115(13):3243-3248, doi: 10.1073/pnas.1720448115.
4. Iljina, M., H. Mazal, P. Goloubinoff, I. Riven, and G. Haran. 2021. Entropic Inhibition: How the Activity of a AAA+ Machine Is Modulated by Its Substrate-Binding Domain. *ACS Chem Biol*. 16(4):775-785, doi: 10.1021/acschembio.1c00156.
5. Kamberaj, H., and A. van der Vaart. 2009. Correlated motions and interactions at the onset of the DNA-induced partial unfolding of Ets-1. *Biophys J*. 96(4):1307-1317, doi: 10.1016/j.bpj.2008.11.019.
6. Damre, M., A. Dayananda, R. A. Varikoti, G. Stan, and R. I. Dima. 2021. Factors underlying asymmetric pore dynamics of disaggregase and microtubule-severing AAA+ machines. *Biophys J*. 120(16):3437-3454, doi: 10.1016/j.bpj.2021.05.027.
7. Grant, B. J., A. P. Rodrigues, K. M. ElSawy, J. A. McCammon, and L. S. Caves. 2006. Bio3d: an R package for the comparative analysis of protein structures. *Bioinformatics*. 22(21):2695-2696, doi: 10.1093/bioinformatics/btl461.
8. Rizo, A. N., J. Lin, S. N. Gates, E. Tse, S. M. Bart, L. M. Castellano, F. DiMaio, J. Shorter, and D. R. Southworth. 2019. Structural basis for substrate gripping and translocation by the ClpB AAA+ disaggregase. *Nat Commun*. 10(1):2393, doi: 10.1038/s41467-019-10150-y.
9. McGibbon, R. T., K. A. Beauchamp, M. P. Harrigan, C. Klein, J. M. Swails, C. X. Hernández, C. R. Schwantes, L. P. Wang, T. J. Lane, and V. S. Pande. 2015. MDTraj: A Modern Open Library for the Analysis of Molecular Dynamics Trajectories. *Biophys J*. 109(8):1528-1532, doi: 10.1016/j.bpj.2015.08.015.
10. Harris, C. R., K. J. Millman, S. J. van der Walt, R. Gommers, P. Virtanen, D. Cournapeau, E. Wieser, J. Taylor, S. Berg, N. J. Smith, R. Kern, M. Picus, S. Hoyer, M. H. van Kerkwijk, M. Brett, A. Haldane, J. F. Del Río, M. Wiebe, P. Peterson, P. Gérard-Marchant, K. Sheppard, T. Reddy, W. Weckesser, H. Abbasi, C. Gohlke, and T. E. Oliphant. 2020. Array programming with NumPy. *Nature*. 585(7825):357-362, doi: 10.1038/s41586-020-2649-2.
11. Tama, F., and Y. H. Sanejouand. 2001. Conformational change of proteins arising from normal mode calculations. *Protein Eng*. 14(1):1-6, doi: 10.1093/protein/14.1.1.
12. Yang, L., G. Song, A. Carriquiry, and R. L. Jernigan. 2008. Close correspondence between the motions from principal component analysis of multiple HIV-1 protease structures and elastic network modes. *Structure*. 16(2):321-330, doi: 10.1016/j.str.2007.12.011.
13. Amadei, A., B. L. de Groot, M. A. Ceruso, M. Paci, A. Di Nola, and H. J. Berendsen. 1999. A kinetic model for the internal motions of proteins: diffusion between multiple harmonic wells. *Proteins*. 35(3):283-292.
14. Girvan, M., and M. E. Newman. 2002. Community structure in social and biological networks. *Proc Natl Acad Sci U S A*. 99(12):7821-7826, doi: 10.1073/pnas.122653799.
15. Scarabelli, G., and B. J. Grant. 2014. Kinesin-5 allosteric inhibitors uncouple the dynamics of nucleotide, microtubule, and neck-linker binding sites. *Biophys J*. 107(9):2204-2213, doi: 10.1016/j.bpj.2014.09.019.

16. Van Wart, A. T., J. Durrant, L. Votapka, and R. E. Amaro. 2014. Weighted Implementation of Suboptimal Paths (WISP): An Optimized Algorithm and Tool for Dynamical Network Analysis. *J Chem Theory Comput*. 10(2):511-517, doi: 10.1021/ct4008603.
17. Bradley, E. L. 1985. "Overlapping Coefficient". In Encyclopedia of Statistical Sciences. John Wiley.
18. Weitzman, M. S. 1970. Measures of overlap of income distributions of white and negro families in the United States. US Bureau of the Census.
19. Mazal, H., M. Iljina, Y. Barak, N. Elad, R. Rosenzweig, P. Goloubinoff, I. Riven, and G. Haran. 2019. Tunable microsecond dynamics of an allosteric switch regulate the activity of a AAA+ disaggregation machine. *Nat Commun*. 10(1):1438, doi: 10.1038/s41467-019-09474-6.
20. Horovitz, A. 1996. Double-mutant cycles: a powerful tool for analyzing protein structure and function. *Fold Des*. 1(6):R121-126, doi: 10.1016/S1359-0278(96)00056-9.
21. Deville, C., K. Franke, A. Mogk, B. Bukau, and H. R. Saibil. 2019. Two-Step Activation Mechanism of the ClpB Disaggregase for Sequential Substrate Threading by the Main ATPase Motor. *Cell Rep*. 27(12):3433-3446.e3434, doi: 10.1016/j.celrep.2019.05.075.
